# Gestational age at birth and type 1 diabetes in childhood and young adulthood: a nationwide register study in Finland, Norway and Sweden

**DOI:** 10.1007/s00125-024-06139-y

**Published:** 2024-04-13

**Authors:** Johanna Metsälä, Kari Risnes, Martina Persson, Riitta Veijola, Anna Pulakka, Katriina Heikkilä, Suvi Alenius, Mika Gissler, Signe Opdahl, Sven Sandin, Eero Kajantie

**Affiliations:** 1 Population Health Unit, Finnish Institute for Health and Welfare, Helsinki, Finland; 2 Department of Clinical and Molecular Medicine, Norwegian University of Science and Technology (NTNU), Trondheim, Norway; 3 Children’s Clinic, St Olav University Hospital, Trondheim, Norway; 4 Department of Medicine, Karolinska Institutet, Stockholm, Sweden; 5 Department of Clinical Science and Education Södersjukhuset, Karolinska Institutet, Stockholm, Sweden; 6 Department of Endocrinology and Diabetology, Sachsska Childrens’ and Youth Hospital, Stockholm, Sweden; 7 Clinical Medicine Research Unit, MRC Oulu, Oulu University Hospital and University of Oulu, Oulu, Finland; 8 Research Unit of Population Health, Faculty of Medicine, University of Oulu, Oulu, Finland; 9 Department of Public Health, University of Turku, Turku, Finland; 10 Centre for Population Health Research, University of Turku and Turku University Hospital, Turku, Finland; 11 Children’s Hospital, University of Helsinki and Helsinki University Hospital, Helsinki, Finland; 12 Department of Knowledge Brokers, Finnish Institute for Health and Welfare, Helsinki, Finland; 13 Academic Primary Health Care Centre, Region Stockholm, Stockholm, Sweden; 14 Department of Molecular Medicine and Surgery, Karolinska Institutet, Stockholm, Sweden; 15 Department of Public Health and Nursing, Norwegian University of Science and Technology (NTNU), Trondheim, Norway; 16 Department of Medical Epidemiology and Biostatistics, Karolinska Institutet, Stockholm, Sweden; 17 Department of Psychiatry, Icahn School of Medicine at Mount Sinai, New York, NY USA; 18 Seaver Autism Center for Research and Treatment at Mount Sinai, New York, NY USA

**Keywords:** Adolescent, Children, Fetal growth, Gestational age, Preterm birth, Type 1 diabetes

## Abstract

**Aims/hypothesis:**

Children and adults born preterm have an increased risk of type 1 diabetes. However, there is limited information on risk patterns across the full range of gestational ages, especially after extremely preterm birth (23–27 weeks of gestation). We investigated the risk of type 1 diabetes in childhood and young adulthood across the full range of length of gestation at birth.

**Methods:**

Data were obtained from national registers in Finland, Norway and Sweden. In each country, information on study participants and gestational age was collected from the Medical Birth Registers, information on type 1 diabetes diagnoses was collected from the National Patient Registers, and information on education, emigration and death was collected from the respective national register sources. Individual-level data were linked using unique personal identity codes. The study population included all individuals born alive between 1987 and 2016 to mothers whose country of birth was the respective Nordic country. Individuals were followed until diagnosis of type 1 diabetes, death, emigration or end of follow-up (31 December 2016 in Finland, 31 December 2017 in Norway and Sweden). Gestational age was categorised as extremely preterm (23–27 completed weeks), very preterm (28–31 weeks), moderately preterm (32–33 weeks), late preterm (34–36 weeks), early term (37–38 weeks), full term (39–41 weeks; reference) and post term (42–45 weeks). HRs and 95% CIs from country-specific covariate-adjusted Cox regression models were combined in a meta-analysis using a common-effect inverse-variance model.

**Results:**

Among 5,501,276 individuals, 0.2% were born extremely preterm, 0.5% very preterm, 0.7% moderately preterm, 4.2% late preterm, 17.7% early term, 69.9% full term, and 6.7% post term. A type 1 diabetes diagnosis was recorded in 12,326 (0.8%), 6364 (0.5%) and 16,856 (0.7%) individuals at a median age of 8.2, 13.0 and 10.5 years in Finland, Norway and Sweden, respectively. Individuals born late preterm or early term had an increased risk of type 1 diabetes compared with their full-term-born peers (pooled, multiple confounder-adjusted HR 1.12, 95% CI 1.07, 1.18; and 1.15, 95% CI 1.11, 1.18, respectively). However, those born extremely preterm or very preterm had a decreased risk of type 1 diabetes (adjusted HR 0.63, 95% CI 0.45, 0.88; and 0.78, 95% CI 0.67, 0.92, respectively). These associations were similar across all three countries.

**Conclusions/interpretation:**

Individuals born late preterm and early term have an increased risk of type 1 diabetes while individuals born extremely preterm or very preterm have a decreased risk of type 1 diabetes compared with those born full term.

**Graphical Abstract:**

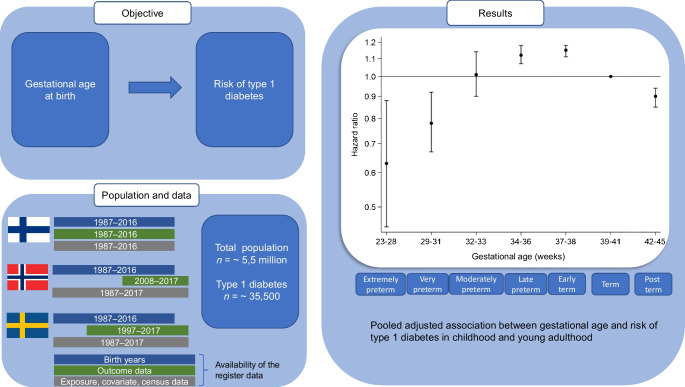

**Supplementary Information:**

The online version of this article (10.1007/s00125-024-06139-y) contains peer-reviewed but unedited supplementary material.


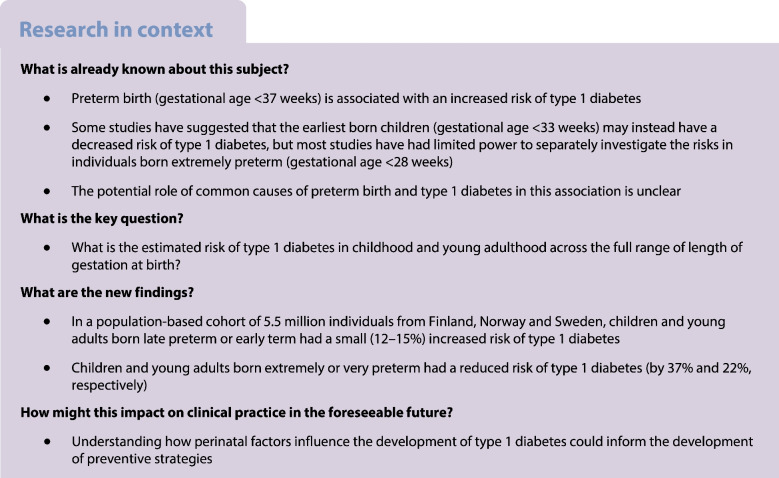


## Introduction

The incidence of type 1 diabetes has increased worldwide over the last few decades, although the increase appears to have levelled off in some high-incidence countries [1]. Genetic factors, namely variation in the HLA region, are involved in the pathogenesis of type 1 diabetes, but the increase in incidence supports an aetiological role of environmental exposures, such as infections [1, 2]. The clinical onset of type 1 diabetes is preceded by an asymptomatic phase, characterised by development of autoantibodies to pancreatic beta cell antigens in genetically susceptible individuals. This preclinical phase can last from months to years, and the first autoantibodies can be detected as early as 6 months of age, peaking at 12–24 months [3]. The appearance of autoantibodies at this early stage in life suggests that environmental factors during the perinatal or early postnatal period may play a role in the underlying pathophysiology.

Preterm birth, before 37 weeks of gestation, is an important early life event with known health consequences [4]. Around 6% of all births occur preterm in the Nordic countries and 10% globally [5]. Several studies have reported that children and adolescents born preterm have a higher risk of type 1 diabetes than those born at term [6–12]. Some studies have suggested that the risk of type 1 diabetes depends on the degree of prematurity: an increased risk among children born late preterm and a decreased risk among those born at the earliest gestational weeks [6–8, 10]. However, most previous studies have had limited statistical power to assess the risks in individuals born extremely preterm (gestational age <28 weeks). Thus, the risk of type 1 diabetes across the full range of gestational ages remains unclear. In addition, the potential impact of the underlying causes of preterm birth, such as pregnancy complications and abnormal fetal growth, on the association between preterm birth and type 1 diabetes is unclear.

Our primary aim was to investigate the risk of type 1 diabetes in childhood, adolescence and early adulthood across the full range of length of gestation at birth. Second, we examined the risk of type 1 diabetes in specific exposure groups: two groups linked to environmental exposures, that is, preterm birth with abnormal fetal growth and preterm birth with maternal hypertensive disorder during pregnancy, and one group linked to inherited risk defined as maternal type 1 diabetes during pregnancy.

## Methods

### Data sources and study population

Our cohort study is based on data from multiple national registers in Finland, Norway and Sweden. Within each country, data were linked using the unique personal identity code provided to every citizen and to permanent residents. The study population, identified from the Medical Birth Registers (MBRs) [13], comprised all individuals born alive between 1 January 1987 and 31 December 2016 and followed to 31 December 2016 in Finland and 31 December 2017 in Norway and Sweden. Information from the MBRs was linked to information on specialised healthcare from the National Patient Registers (NPRs) and information on education, emigration and death from the respective statistical offices and national registers.

### Type 1 diabetes

Information on type 1 diabetes was obtained from the NPRs: the Finnish Care Register for Health Care [14], the Norwegian Patient Registry [15] and the Swedish Patient Register [16]. In each country, type 1 diabetes was defined based on the first recorded ICD code for type 1 diabetes: E10 (10th revision; https://icd.who.int/browse10/2019/en) or 250.*1 or 250.*3 (9th revision, used in Finnish data only; http://www.icd9data.com/2007/Volume1/default.htm), either as a main or secondary diagnosis at inpatient or outpatient visits.

The NPR data were available from 1 January 1987 to 31 December 2016 in Finland and from 1 January 2008 to 31 December 2017 in Norway. In Sweden, we restricted the outcome assessment period from 1 January 1997 to 31 December 2017 because type 1 diabetes has been distinguishable from other diabetes types only since the introduction of ICD-10 coding in 1997.

### Gestational age

Information on gestational age at birth in completed weeks was extracted from the MBRs, with the following order of preference for estimation method (as available): date of embryo transfer in cases of assisted reproduction, ultrasonography, last menstrual period or clinical examination [17, 18]. Gestational age was categorised as extremely preterm (23–27 completed weeks), very preterm (28–31 weeks) moderately preterm (32–33 weeks), late preterm (34–36 weeks), early term (37–38), full term (39–41 weeks) and post term (42–45 weeks).

### Covariates

We adjusted the estimates for child, maternal and paternal characteristics previously shown to be associated with preterm birth [19, 20] and type 1 diabetes [1]. The potentially confounding factors were child’s sex (male/female, registered at birth), birth year (1987–1989, 1990–1999, 2000–2009, 2010–2016) and birthweight *z* score (categorised as <−2, from −2 to <−1, from −1 to <0, from 0 to <1, from 1 to <2 and ≥2). As different growth references produce large differences in classification of small-for-gestational-age infants, particularly at low numbers of gestational weeks [21], we calculated birthweight *z* scores based on two growth references that use alternative approaches: a birthweight reference from Sankilampi et al [22] and an intrauterine reference from Maršál et al [23]. Mothers’ characteristics were measured at delivery—age (treated as continuous), parity (number of previous livebirths: 0, 1 or ≥2) and highest level of educational attainment (low, corresponding to International Standard Classification of Education [ISCED] [24] classes 0–2; intermediate: ISCED classes 3–5; or high: ISCED classes 6–8)—or during pregnancy: diabetes (type 1, type 2/other pre-pregnancy diabetes and gestational diabetes), hypertensive disorders (chronic hypertension, gestational hypertension, pre-eclampsia and eclampsia) and Caesarean section (yes/no). Data sources, ICD codes and predefined MBR variables used to define maternal health conditions in each country are presented in electronic supplementary material (ESM) Table 1. Fathers’ characteristics comprised age (continuous) and educational level (categorised as for mothers’ education) at child’s birth, type 1 diabetes (in Finland only) and country of birth (in Finland only, categorised as Finland, other high-income country or any other country). Information on race or ethnicity was not available in this study.

### Statistical analysis

The associations between gestational age and type 1 diabetes were estimated using HRs and 95% CIs obtained from Cox proportional hazards regression models. Participants were followed from either birth or start of NPR data availability, whichever occurred last, until death, emigration, first record of type 1 diabetes diagnosis or end of follow-up, whichever occurred first. The main analyses were conducted using seven categories of gestational age, but in all subgroup and sensitivity analyses the extremely and very preterm birth groups were combined (<32 weeks). The proportional hazards assumption was evaluated by visual inspection of log–log plots, and assumptions were met in all models. To examine whether our findings could represent false positives from multiple testing, we calculated *p* values corrected for six comparisons using the Holm procedure [25]. All analyses were first conducted within each country and then combined across all countries using common-effect inverse-variance models [26]. We performed a combined analysis only when data from all countries were available.

In the main analyses, we fitted an unadjusted model and four covariate-adjusted models that built on each other. Adjusted model 1 included child’s sex and birth year, whereas model 2 additionally included child’s birthweight *z* score (based on Sankilampi et al [22]) and maternal factors at delivery (age, education level, parity, diabetes during pregnancy and hypertensive disorder during pregnancy). Model 3 additionally included paternal factors, that is, father’s age and education level at the birth of the child, and model 4 additionally included father’s type 1 diabetes status and country of origin (available only in the Finnish data). We considered model 2 the main model.

To examine whether fetal growth (approximated by birthweight *z* score), type 1 diabetes in the mother or hypertensive disorders during pregnancy modified the association between gestational age and type 1 diabetes in offspring, we stratified the analyses by levels of these covariates using one combined reference category.

We conducted sensitivity analyses to examine the extent to which our findings were influenced by: (1) death as a competing event; (2) improvements in survival rate and neonatal care during the long follow-up period; (3) limited availability of outcome data in Norway and Sweden; (4) residual confounding from unmeasured familial confounders or mode of delivery; or (5) operationalisation of birthweight *z* score and gestational age.

We estimated death as a competing event and potential sources of informative censoring using cause-specific hazards of death and cause-specific hazards of type 1 diabetes, with different assumptions for the incidence of type 1 diabetes among those who died had they survived: (1) 0.2 times lower, (2) equal to, (3) 1.5 times higher, (4) two times higher and (5) 2.5 times higher incidence of type 1 diabetes than observed in those who did not die. Furthermore, we estimated cause-specific cumulative incidence functions for type 1 diabetes in categories of gestational age. Impacts of improvements in survival rate and neonatal care and limited availability of outcome data were investigated by restricting the study population in the main analyses, first, to those born and followed since 1997 in Sweden and Finland and, then, to those born and followed since 2008 in Norway, Sweden and Finland. To evaluate potential confounding by unmeasured shared familial factors, we conducted a sibling analysis of full siblings (same mother and father), estimating the within-family risk of type 1 diabetes in a stratified Cox regression with families as strata. Further, we ran an additional adjusted model by including Caesarean section in the main model (model 2). To compare the results using birthweight *z* scores based on different growth charts, we reran the analyses using growth curves from Maršál et al [23]. To investigate gestational age week by week we treated each completed week as its own category with week 40 as the reference. Because there were no or few individuals with type 1 diabetes in some gestational weeks, weeks 23–28 and 43–44 were combined to obtain pooled estimates.

### Ethical approval

This study was approved by the relevant register authorities in each country: the Institutional Review Board of the Finnish Institute for Health and Welfare (THL 1960/6.02.00/2018), the Central Norway Regional Committee for Medical Research Ethics (2018/32) and the Swedish Ethical Review Board in Stockholm (2017/1875-31/1). In Finland and Sweden, informed consent is not required for the use of pseudonymised register data for research purposes. In Norway, the Central Norway Regional Committee for Medical Research Ethics gave an exemption from the requirement to obtain informed consent as part of the ethics approval.

## Results

We identified 5,631,429 individuals born alive between 1987 and 2016 to mothers whose country of origin was Finland, Norway or Sweden (country-specific information is provided in ESM Fig. 1). We included only children whose mother was born in the respective Nordic country to reduce confounding by ethnicity. We excluded individuals who had missing information on gestational age; had a gestational age <23 weeks or >45 weeks; had implausible combinations of gestational age and birthweight (birthweight *z* score <−6, birthweight <300 g or birthweight *z* score >4 in those born earlier than 37 weeks); had died or emigrated at or before the start of follow-up; or had missing information for any of the covariates. Complete covariate information was available for 5,501,276 (97.7%) individuals, who were included in the analyses (ESM Fig. 1).

In total, 10,865 (0.2%) participants were born extremely preterm (23–27 weeks), 20,890 (0.4%) very preterm (28–31 weeks), 40,645 (0.7%) moderately preterm (32–33 weeks), 233,368 (4.2%) late preterm (34–36 weeks), 976,342 (17.7%) early term (37–38 weeks), 3,843,280 (69.9%) full term (39–41 weeks) and 366,886 (6.7%) post term (42–25 weeks). During a median of 15.6, 10.0 and 15.7 years of follow-up, 12,326 (0.8%), 6364 (0.5%) and 16,856 (0.7%) individuals had a type 1 diabetes diagnosis recorded in Finland, Norway and Sweden, respectively, comprising a total of 35,546 individuals with a type 1 diabetes diagnosis. The median (IQR) age at the first record of a type 1 diabetes diagnosis was 8.2 (4.5, 12.2), 13.0 (8.9, 17.1) and 10.5 (6.6, 14.4) years in Finland, Norway and Sweden, respectively. Key characteristics of the study population by gestational age categories and country are presented in Table 1 and full characteristics of the study population are provided in ESM Table 2.

**Table 1:** Characteristics of the study population

Characteristic	**Gestational age category (completed weeks)** Country: *n* (%)													
	**Extremely preterm (23–27 weeks)** FI: 3343 (0.2) NO: 2480 (0.2) SE: 5042 (0.2)		**Very preterm (28–31 weeks)** FI: 8062 (0.5) NO: 7926 (0.6) SE: 13,902 (0.6)		**Moderately preterm (32–33 weeks)** FI: 10,912 (0.7) NO: 10,894 (0.8) SE: 18,839 (0.8)		**Late preterm (34–36 weeks)** FI: 65,956 (4.0) NO: 60,062 (4.5) SE: 107,350 (4.3)		**Early term (37–38 weeks)** FI: 295,705 (17.9) NO: 214,271 (16.0) SE: 466,366 (18.6)		**Full term (39–41 weeks)** FI: 1,196,059 (72.3) NO: 924,216 (69.1) SE: 1,723,005 (68.6)		**Post term (42–45 weeks)** FI: 73,359 (4.4) NO: 117,840 (8.8) SE: 175,687 (7.0)	
	*n*	%	*n*	%	*n*	%	*n*	%	*n*	%	*n*	%	*n*	%
Finland														
Sex, male	1815	54.3	4466	55.4	6096	55.9	35,953	54.5	156,745	53.0	601,976	50.3	37,700	51.4
Birth year														
1999 or earlier	1508	45.1	3591	44.5	4803	44.0	29,978	45.5	137,696	46.6	558,276	46.7	33,652	45.9
2000 or after	1835	54.9	4472	55.5	6109	56.0	35,978	54.5	158,009	53.4	637,783	53.3	39,707	54.1
Type 1 diabetes	12	0.4	45	0.6	90	0.8	557	0.8	2587	0.9	8533	0.7	502	0.7
Age, mother (years), mean (SD)	30.2	(5.7)	30.1	(5.7)	29.9	(5.7)	29.7	(5.6)	29.7	(5.4)	29.2	(5.2)	29.0	(5.2)
Diabetes, mother	199	6.0	769	9.5	1207	11.1	8469	12.8	36,297	12.3	92,623	7.7	4337	5.9
Hypertensive disorder, mother	525	15.7	2154	26.7	2524	23.1	11,527	17.5	31,994	10.8	68,202	5.7	2927	4.0
Follow-up (years), median (IQR)	8.5	(0.2, 18.7)	14.3	(6.5, 21.6)	14.6	(7.3, 22.0)	15.3	(7.5, 22.7)	15.7	(7.9, 23.0)	15.7	(7.8, 23.2)	15.4	(8.1, 23.1)
Age at end of follow-up (years), median (IQR)	8.5	(0.2, 18.7)	14.3	(6.5, 21.6)	14.6	(7.3, 22.0)	15.3	(7.5, 22.7)	15.7	(7.9, 23.0)	15.7	(7.8, 23.2)	15.4	(8.1, 23.1)
Norway														
Sex, male	1290	52.0	4337	54.7	5929	54.4	32,173	53.6	110,484	51.6	469,223	50.8	63,061	53.5
Birth year														
1999 or earlier	790	31.9	3001	37.9	4266	39.2	24,036	40.0	78,852	36.8	384,619	41.6	71,825	61.0
2000 or after	1690	68.1	4925	62.1	6628	60.8	36,026	60.0	135,419	63.2	539,597	58.4	46,015	39.0
Type 1 diabetes	6	0.2	24	0.3	55	0.5	320	0.5	1160	0.5	4253	0.5	546	0.5
Age, mother (years), mean (SD)	30.1	(5.5)	29.7	(5.5)	29.7	(5.4)	29.5	(5.4)	29.8	(5.3)	29.4	(5.0)	28.7	(5.0)
Diabetes, mother	51	2.1	247	3.1	373	3.4	2231	3.7	6990	3.3	11,723	1.3	489	0.4
Hypertensive disorder, mother	456	18.4	2014	25.4	2316	21.3	9507	15.8	17,964	8.4	39,441	4.3	4007	3.4
Follow-up (years), median (IQR)	10.0	(6.8, 10.0)	10.0	(8.4, 10.0)	10.0	(8.6, 10.0)	10.0	(8.5, 10.0)	10.0	(8.0, 10.0)	10.0	(8.1, 10.0)	10.0	(10.0, 10.0)
Age at end of follow-up (years), median (IQR)	13.7	(7.0, 19.9)	15.0	(8.5, 21.5)	15.1	(8.8, 22.0)	15.3	(8.6, 22.5)	14.5	(8.1, 21.7)	15.5	(8.3, 22.9)	20.5	(13.4, 25.5)
Sweden														
Sex, male	2749	54.5	7578	54.5	10,272	54.5	57,475	53.5	236,277	50.7	875,089	50.8	100,661	57.3
Birth year														
1999 or earlier	1638	32.5	6086	43.8	8514	45.2	49,404	46.0	208,443	44.7	785,046	45.6	79,876	45.5
2000 or after	3404	67.5	7816	56.2	10,325	54.8	57,946	54.0	257,923	55.3	937,959	54.4	95,811	54.5
Type 1 diabetes	17	0.3	79	0.6	123	0.7	865	0.8	3558	0.8	11,189	0.6	1025	0.6
Age, mother (years), mean (SD)	30.6	(5.6)	30.4	(5.4)	30.2	(5.4)	30.1	(5.4)	30.4	(5.3)	30.0	(5.0)	30.2	(5.0)
Diabetes, mother	96	1.9	361	2.6	445	2.4	2719	2.5	8573	1.8	11,833	0.7	706	0.4
Hypertensive disorder, mother	61	1.2	198	1.4	188	1.0	842	0.8	2531	0.5	5876	0.3	468	0.3
Follow-up (years), median (IQR)	11.1	(4.4, 19.6)	15.3	(8.0, 21.0)	15.7	(8.1, 21.0)	16.0	(8.5, 21.0)	15.6	(8.3, 21.0)	15.8	(8.1, 21.0)	16.0	(8.5, 21.0)
Age at end of follow-up (years), median (IQR)	11.2	(4.4, 19.7)	15.5	(8.0, 23.8)	15.9	(8.2, 24.0)	16.3	(8.5, 24.4)	15.8	(8.4, 24.2)	16.0	(8.2, 24.2)	16.2	(8.5, 24.1)

FI, Finland; NO, Norway; SE, Sweden

**Fig. 1:**
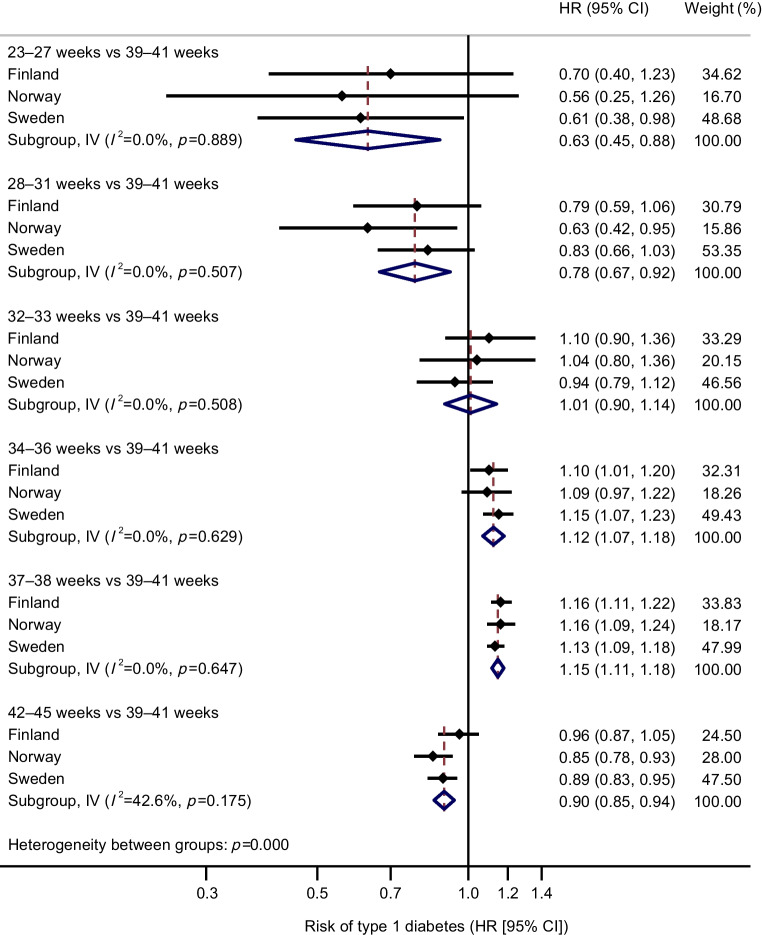
Association between gestational age and risk of type 1 diabetes in offspring in Finland, Norway and Sweden. HRs are adjusted for child’s sex, birth year and birthweight *z* score and mother’s age, education level, parity, diabetes during pregnancy and hypertensive disorder during pregnancy (model 2). IV, inverse variance; *I*^2^, I-square statistic for heterogeneity

Analyses combining estimates for the three countries showed higher risks of type 1 diabetes in childhood and young adulthood among individuals born late preterm (34–36 weeks) and early term (37–38 weeks) than among those born full term (39–41 weeks) (pooled, adjusted HR 1.12, 95% CI 1.07, 1.18; 1.15, 95% CI 1.11, 1.18, respectively) after adjustment for child’s sex, birth year and birthweight *z* score and mother’s age, education level, parity, diabetes status and hypertensive disorder during pregnancy (Fig. 1). Extremely preterm (23–27 weeks) (pooled, adjusted HR 0.63, 95% CI 0.45, 0.88) and very preterm (28–31 weeks) (pooled, adjusted HR 0.78, 95% CI 0.67, 0.92) births were, in turn, associated with a decreased risk of type 1 diabetes compared with full-term births. Associations were relatively consistent across countries and adjustments (ESM Table 3), and the estimates were statistically significant at a Holm-adjusted alpha level of 0.05 (ESM Table 4).

The pattern of an increased risk of type 1 diabetes in childhood and young adulthood in individuals born late preterm (34–36 weeks) or early term (37–38 weeks) and a decreased risk of type 1 diabetes in individuals born extremely preterm (23–27 weeks) or very preterm (28–31 weeks) was present among children with birthweight *z* scores of ≥0, but not among those with lower birthweight *z* scores, after adjustment for child’s sex, birth cohort and mother’s age, education level, parity, diabetes during pregnancy and hypertensive disorders during pregnancy [pooled estimates in Table 2; country-specific estimates and numbers of individuals in ESM Tables 5 and 6, respectively]. In total, 32,678 (0.6%) mothers had type 1 diabetes and 1118 (3.4%) of their offspring were diagnosed with type 1 diabetes. We observed no clear pattern in the risk of type 1 diabetes by gestational age categories among offspring of mothers with type 1 diabetes, while the risk pattern among offspring of mothers without type 1 diabetes resembled that of the whole population (pooled estimates in Table 3; country-specific results in ESM Table 7). The risk pattern for type 1 diabetes was similar between offspring of mothers with a hypertensive disorder during pregnancy and offspring of mothers without a hypertensive disorder during pregnancy (pooled estimates in Table 4; country-specific results in ESM Table 8).

**Table 2:** Pooled association of combinations of gestational age and birthweight *z* scores with risk of type 1 diabetes in offspring

Gestational age category (weeks)	**Birthweight** ***z* score category**^a^ Country: type 1 diabetes/total, *n*																	
	**<−2** FI: 245/46,056 NO: 186/42,592 SE: 316/51,213			**−2 to <−1** FI: 1627/239,647 NO: 837/199,026 SE: 1713/282,483			**−1 to <0** FI: 4167/592,696 NO: 2081/472,254 SE: 5195/810,299			**0 to <+1** FI: 4102/528,260 NO: 2099/418,493 SE: 5919/836,001			**+1 to <+2** FI: 1677/198,181 NO: 890/161,730 SE: 2772/386,606			**≥+2** FI: 508/48,556 NO: 271/43,594 SE: 941/116,589		
	HR^b^	95% CI	*I*^2^ (%)	HR^b^	95% CI	*I*^2^ (%)	HR^b^	95% CI	*I*^2^ (%)	HR^b^	95% CI	*I*^2^ (%)	HR^b^	95% CI	*I*^2^ (%)	HR^b^	95% CI	I^2^ (%)
<32	0.55	0.34, 0.89	0.0	0.62	0.41, 0.96	0.0	0.93	0.69, 1.23	51.6	0.78	0.59, 1.02	0.0	0.58	0.39, 0.85	0.0	0.80	0.53, 1.22	0.0
32–33	0.68	0.44, 1.08	0.0	0.62	0.41, 0.94	0.0	1.15	0.92, 1.44	0.0	0.95	0.75, 1.21	0.0	0.96	0.71, 1.30	53.7	1.45	1.06, 1.98	0.0
34–36	0.75	0.60, 0.94	74.5	0.94	0.82, 1.08	3.2	1.02	0.93, 1.12	0.0	1.18	1.08, 1.29	0.0	1.21	1.08, 1.36	72.2	1.19	1.02, 1.39	0.0
37–38	0.88	0.76, 1.01	0.0	0.96	0.89, 1.03	0.0	1.07	1.02, 1.12	0.0	1.17	1.12, 1.23	0.0	1.23	1.16, 1.30	75.4	1.16	1.05, 1.28	0.0
39–41	0.83	0.75, 0.92	64.0	0.90	0.86, 0.93	55.8	0.93	0.90, 0.96	0.0	1.00	[Reference]		1.03	0.99, 1.08	0.0	1.06	0.99, 1.14	0.0
42–45	0.75	0.54, 1.04	61.8	0.81	0.71, 0.91	42.5	0.83	0.77, 0.90	0.0	0.90	0.84, 0.97	0.0	0.92	0.82, 1.03	19.9	0.84	0.66, 1.06	0.0

FI, Finland; NO, Norway; SE, Sweden; *I*^2^, I-square statistic for heterogeneity

^a^Birthweight *z* scores calculated according to Sankilampi et al [22]

^b^HRs are adjusted for child’s sex and birth year and mother’s age, parity, education level, diabetes during pregnancy and hypertensive disorder during pregnancy (model 2 without birthweight *z* score)

**Table 3:** Pooled association of combination of gestational age and mother’s type 1 diabetes status with risk of type 1 diabetes in offspring

Gestational age category (weeks)	**Maternal type 1 diabetes**^a^ Country: type 1 diabetes/total, *n*					
	**No** FI: 12,044/1,646,018 NO: 2661/815,337 SE: 16,106/2,488,805			**Yes** FI: 282/7378 NO: 86/3914 SE: 750/21,386		
	HR^b^	95%CI	*I*^2^ (%)	HR^b^	95%CI	*I*^2^ (%)
<32	0.73	0.62, 0.85	0.0	5.33	3.44, 8.27	0.0
32–33	0.99	0.87, 1.14	0.0	6.99	4.96, 9.84	34.0
34–36	1.15	1.08, 1.21	0.0	5.71	4.90, 6.66	0.0
37–38	1.16	1.13, 1.19	0.0	5.78	5.27, 6.35	42.3
39–41	1.00	[Reference]		5.10	4.63, 5.62	28.8
42–45	0.90	0.86, 0.95	11.4	4.33	2.76, 6.80	0.0

FI, Finland; NO, Norway; SE, Sweden; *I*^2^, I-square statistic for heterogeneity

^a^In Norway, analyses were restricted to those born in 1999 or later, as data on maternal pre-pregnancy diabetes subtypes are available only from 1999

^b^HRs were adjusted for child’s sex, birth year and birthweight *z* score and mother’s age, parity, education level and hypertensive disorder during pregnancy (model 2 without diabetes during pregnancy)

**Table 4:** Pooled association of combination of gestational age and maternal hypertensive disorder during pregnancy with risk of type 1 diabetes in offspring

Gestational age category (weeks)	**Maternal hypertensive disorder during pregnancy** Country: type 1 diabetes/total, *n*					
	**No** FI: 11,379/1,533,543 NO: 5994/1,261,984 SE: 16,779/2,500,027			**Yes** FI: 947/119,853 NO: 370/75,705 SE: 77/10,164		
	HR^a^	95% CI	*I*^2^ (%)	HR^a^	95% CI	*I*^2^ (%)
<32	0.77	0.66, 0.89	25.2	0.69	0.44, 1.07	0.0
32–33	1.02	0.90, 1.15	0.0	1.02	0.72, 1.45	0.0
34–36	1.13	1.08, 1.19	0.0	1.09	0.93, 1.28	0.0
37–38	1.15	1.12, 1.19	0.0	1.15	1.03, 1.27	42.2
39–41	1.00	[Reference]		1.09	1.01, 1.18	0.0
42–45	0.90	0.86, 0.94	44.4	0.89	0.65, 1.21	0.0

FI, Finland; NO, Norway; SE, Sweden; *I*^2^, I-square statistic for heterogeneity

^a^HRs are adjusted for child’s sex, birth year and birthweight *z* score and mother’s age, parity, education level and diabetes during pregnancy (model 2 without hypertensive disorder during pregnancy)

Sensitivity analyses showed that low gestational age was much more strongly associated with risk of death than with risk of type 1 diabetes in childhood and young adulthood (ESM Table 9), and that a positive dependence between the two outcomes could reverse the association between extremely preterm birth and type 1 diabetes (ESM Table 10). Cause-specific cumulative incidence for type 1 diabetes by gestational age is presented in ESM Fig. 2. Restricting the study population to individuals with information on type 1 diabetes available since birth gave results that were similar to the main results (ESM Table 11), as did the sibling analysis (74–76% of the total cohort in each country; pooled and country-specific results in ESM Table 12). For example, within families, the pooled adjusted HR for type 1 diabetes associated with extremely or very preterm birth (<32 weeks) was 0.82 (95% CI 0.62, 1.09), while the corresponding result in the main analysis was 0.75 (95% CI 0.65, 0.87). Choice of reference for calculation of birthweight *z* scores had no substantial effect on the results of either the main analysis (ESM Table 13) or the subgroup analysis (ESM Table 14). Additional adjustment for Caesarean section had a negligible effect on the risk estimates (ESM Table 3). In the week-by-week analysis, the highest pooled HR was observed for gestational week 37 (1.24, 95% CI 1.18, 1.30) with a gradual decrease in the HR in both lower and higher gestational weeks when compared with gestational week 40 (ESM Fig. 3, ESM Table 15).

## Discussion

In this population-based register study of over 5.5 million people in three Nordic countries, the direction of the association between gestational age at birth and risk of type 1 diabetes differed between gestational age categories. Compared with those born full term, the risk was higher for those born late preterm (34–36 weeks) or early term (37–38 weeks) and lower for those born extremely preterm (23–27 weeks) or very preterm (28–31 weeks). Subgroup analyses suggested that the association between late preterm (34–36 weeks) or early term (37–38 weeks) birth and increased risk of type 1 diabetes may be stronger among those with more rapid fetal growth.

Overall, the magnitude of the risk of type 1 diabetes was small but consistent across countries, adjustments and sensitivity analyses. Further, it is important to note that the higher risk was not only limited to preterm birth but also extended into early term (37–38 weeks) birth, which accounts for approximately 18% of all babies. Thus, even a small or modest increase in the risk of type 1 diabetes may have significant public health implications.

A number of studies have reported an increased risk of type 1 diabetes in children and adults born preterm (gestational age <37 weeks) [6–12], although only a few studies have investigated the risk across the entire range of gestational age. Studies from Sweden and Finland have reported an increased risk of type 1 diabetes in later preterm and early term infants and a decreased risk in those born at the earliest gestational weeks [6–8, 10]. Our results are in line with these findings, although the studies differed in their categorisation of gestational age and data sources used to identify type 1 diabetes. For example, in most studies, the lowest gestational age category was <33 weeks, which includes children born extremely preterm (<28 weeks) and very preterm (28–31 weeks) and also partly those born moderately preterm (32–33 weeks), whereas we investigated these three groups separately. As far as we know, only Crump et al [7] investigated extremely preterm birth separately, and their result (HR 0.51, 95% CI 0.32, 0.81) for risk of type 1 diabetes at age <18 years is very close to our pooled result (HR 0.63, 95% CI 0.45, 0.88), but their study years partly overlap with those in the present study.

The mechanism underlying the association between gestational age at birth and risk of type 1 diabetes is unclear. This association likely reflects the interplay between genetic and pre- and postnatal environmental risk factors. Babies born at different gestational ages may face very different pre- and postnatal environments, including exposure and sensitisation to allergens and other foreign proteins, administration of certain medications (e.g. glucocorticoids and antibiotics) and feeding regimens, which may affect, among other things, growth [27] and the gut microbiome [28]. The environment may also differ according to the cause of preterm birth, as well as with neonatal conditions associated with varying degrees of prematurity. It seems likely that the timing of the adverse exposure in relation to beta cell maturation affects the risk of later type 1 diabetes. The fetal allocation of beta cells is completed at the end of the second/start of the third trimester, and beta cell mass increases thereafter and continues to increase for several years after birth [29]. However, the mechanisms regulating beta cell maturation and the potential impact of environmental exposures during different periods of fetal development are largely unknown.

The large prospective TEDDY study, which follows children who are genetically susceptible to type 1 diabetes from birth, recently reported that higher gestational age-adjusted birthweight and a higher rate of weight gain in infancy were associated with an increased risk of islet autoimmunity, a precursor to type 1 diabetes [30]. Our finding of an increased risk of type 1 diabetes in late preterm and early term children might be associated with the possibility that such slightly preterm children are prone to overnutrition and rapid weight gain [31, 32]. This may challenge insulin-producing beta cells and cause endoplasmic reticulum stress [33, 34]. This, in turn, may lead to the exposure of beta cell antigens to the immune system, which can result in islet autoimmunity in susceptible individuals.

The mechanism for our observation of a decreased, rather than an increased, risk of type 1 diabetes in the most preterm born individuals is unknown. However, in addition to the possibility of informative censoring affecting our observation, there are several other potential explanations. Data from experimental and clinical studies suggest a protective effect of prenatally administered exogenous glucocorticoids on type 1 diabetes risk in very preterm infants [35, 36]. In addition, upregulation of the hypothalamic–pituitary–adrenal (HPA) axis occurs in very preterm infants, which has been suggested as a potential link between very preterm birth and glucose regulation [37]. Upregulation of the HPA axis may be less pronounced among individuals born at later gestational weeks. Further, children born extremely and very preterm are exposed at an immature stage of development to a non-sterile environment with a range of pathogens that not only are associated with high infection rates but also alter the development of microbiota. Altered microbiota may, in turn, impact their immunological responses, including those of an autoimmune nature, and hence reduce the risk of atopic and autoimmune responses. Early programming of the immune system in the earliest born individuals is supported by the observation that adults born preterm and at very low birthweight have lower rates of atopy, a predisposition to respond immunologically to allergens, than their counterparts born at term [38]. Programming of the immune system may therefore affect the predisposition of earliest born individuals to develop immune-mediated conditions, such as type 1 diabetes and allergies.

The key strengths and limitations of our study are both related to the routinely collected administrative data investigated. We combined estimates from nationwide data from three Nordic countries, and the total of 5.5 million individuals, including over 35,000 individuals with type 1 diabetes, provided reasonable statistical power to investigate the risk of type 1 diabetes across the narrow categories of gestational age, including among individuals born extremely preterm (23–27 weeks). A rich set of data including information on several maternal, paternal and child characteristics allowed us to thoroughly investigate the impact of these factors on the association between gestational age and risk of type 1 diabetes. The data from the Nordic registers are, in general, of high quality [14–16, 39, 40]. Although the validity of paediatric type 1 diabetes diagnoses from the NPRs has not been specifically investigated, the NPRs are likely to capture most cases of type 1 diabetes with onset in childhood. In all three countries included in the study, type 1 diabetes in children is diagnosed by specialists in paediatrics; children are typically hospitalised for approximately 1 week after diagnosis and are subsequently regularly followed up by paediatric diabetologists throughout childhood. As the methods for ascertaining gestational age have changed over the study period (see, for example, [39] for Sweden), some misclassification of gestational age is possible. However, as our findings did not change substantially in the sensitivity analysis comparing different time periods, we believe that any potential bias would be minimal.

Although we were able to include several covariates in our analyses, register data, originally collected for administrative purposes, are missing some key covariates, such as HLA type of offspring. Further, we were unable to use data on some potential confounders, such as maternal pre-pregnancy BMI, as they were not available in the registers for a sufficient proportion of individuals. In addition, due to changes in recoding practices in the MBRs and ICD versions over time, some of our covariates, such as mother’s diabetes status, did not have constant definitions over the whole study period. Our results remained similar in comparisons between full siblings, suggesting that the associations found are not due to confounding by shared family factors. However, it is possible that our findings were influenced by residual confounding from other unknown or unmeasured confounders, for example prenatal infections. We were unable to further investigate potential mechanisms underpinning the observed associations, as we did not have information on important postnatal risk factors for type 1 diabetes, such as childhood growth rates, infections or diet [1]. Accounting for death as a competing event through cumulative incidence curves showed a similar risk pattern as in the main analyses. However, sensitivity analyses testing different scenarios of dependency between competing outcomes showed that our findings on extremely preterm birth may be vulnerable to positive dependence and, thus, need to be interpreted with caution. Despite combining results from three large datasets, our subgroup and sensitivity analyses had limited power in small subgroups. We included both men and women in the study and considered sex as a confounder in the analyses. As we were not aware of any theories suggesting sex differences regarding gestational age and risk of type 1 diabetes, and previous research has reported no evidence of sex-interaction [6, 7], we did not include assessment of sex differences in our analyses. As the current results and many of the previous findings stem from Nordic populations, the generalisability of our findings to other populations with different genetic risks and different levels of obstetric and neonatal care remains uncertain.

In conclusion, our findings from nationwide data from Finland, Norway and Sweden consistently show that children born late preterm and early term have increased rates of type 1 diabetes, while the rates are reduced among children born very or extremely preterm. Future studies should acknowledge that children born preterm are not a homogeneous group in terms of their risk of type 1 diabetes. Our results also underline the relevance of perinatal history in risk assessment for type 1 diabetes.

### Supplementary Information

Below is the link to the electronic supplementary material.

Supplementary file1 (PDF 1171 KB)

## Data Availability

Data protection laws do not permit sharing of the data used in this study. Access to the Finnish data can be requested from the Finnish Social and Health Data Permit Authority (Findata; www.findata.fi). Access to the Norwegian data can be requested from the Norwegian Health Data service (www.helsedata.no) and Statistics Norway (www.ssb.no) but requires ethical approval and institutional data protection assessment. Access to the Swedish data can be requested from the National Board of Health and Welfare (www.socialstyrelsen.se) and Statistics Sweden (www.scb.se).

## Abbreviations

ISCED: International Standard Classification of Education
MBR: Medical Birth Register
NPR: National Patient Register
