# Supplementary material for: Gestational age at birth and type 1 diabetes in childhood and young adulthood: a nationwide register study in Finland, Norway and Sweden
Source: Diabetologia. 2024 Apr 13;67(7):1315–27. doi: 10.1007/s00125-024-06139-y (PMC11153267; doi:10.1007/s00125-024-06139-y)
Supplement: Supplementary file 1 — Supplementary file1 (PDF 1171 KB) [file 125_2024_6139_MOESM1_ESM.pdf]

Electronic Supplementary Materials (ESM)

Gestational age at birth and type 1 diabetes in childhood and young adulthood: a nationwide register study in Finland, Norway, and Sweden

Metsälä J, Risnes K, Persson M, Veijola R, Pulakka A, Heikkilä K, Alenius S, Gissler M, Opdahl S, Sandin S, Kajantie E

Table of contents

ESM Table 1 Data sources and ICD codes used in the definition of mother's diabetes and hypertensive disorders during pregnancy in Finland, Norway, and Sweden

ESM Table 2 Full characteristics of the study population by gestational age categories

ESM Table 3 Association between gestational age and the risk of type 1 diabetes – country-specific unadjusted and adjusted results

ESM Table 4 Corrected p-values of the main analyses according to Holm procedure

ESM Table 5 Country-specific associations between combinations of gestational age and birthweight z score and the risk of type 1 diabetes

ESM Table 6 Country-specific total number and number of individuals with type 1 diabetes by gestational age and birthweight z score categories

ESM Table 7 Country-specific associations between combination of gestational age and mother's type 1 diabetes status and risk of type 1 diabetes in offspring

ESM Table 8 Country-specific associations between combination of gestational age and maternal hypertensive disorders during pregnancy and risk of type 1 diabetes in offspring

ESM Table 9 Cause-specific hazards of type 1 diabetes and death and scenarios for extreme informative censoring

ESM Table 10 Association between gestational age and the risk of type 1 diabetes – country-specific and pooled adjusted estimates from analyses investigating death as potential source of informative censoring with different assumptions

ESM Table 11 Association between gestational age and the risk of type 1 diabetes. Comparison of the results from the main and sensitivity analysis of different time periods according to the start of the availability of type 1 diabetes diagnosis data in Sweden (since 1997) and in Norway (since 2008)

ESM Table 12 Country-specific and pooled associations between gestational age and the risk of type 1 diabetes from the sibling analyses

ESM Table 13 Country-specific and pooled associations between gestational age and the risk of type 1 diabetes birth using weight z score calculated according to Maršál reference

ESM Table 14 Country-specific and pooled associations between combinations of gestational age and birthweight z score according to Maršál reference and the risk of type 1 diabetes

ESM Table 15 Country-specific associations between gestational age week by week and the risk of type 1 diabetes

ESM Fig. 1 Flow chart of the study participants

ESM Fig. 2 Cause-specific cumulative incidence of type 1 diabetes in gestational age categories

ESM Fig. 3 Country-specific associations between gestational age by completed weeks and the risk of type 1 diabetes

ESM Table 1 Data sources and ICD codes used in the definition of mother's diabetes and hypertensive disorders during pregnancy in Finland, Norway and Sweden

**Variable name**<br>Country	**Definition/categories of the variables**	**Data source**	**ICD codes/pre-defined variables used in the definition**	**Data extraction period / data availability if not the full study period**
**Mother's diabetes**				
Finland	Type 1 diabetes	NPR, MBR	ICD-10: E10, O24.0<br>ICD-9: 250*B<br>ICD8: 250*	From the NPR data ICD-codes E10, 250*B and 250* that were recoded before child's date of birth were used in the definition<br><br>ICD-8 codes were used to define the date of first diagnosis, but subsequent ICD-9 codes were used to define the diabetes type [a]
	Type 2/other pre-pregnancy diabetes	NPR, MBR	ICD-10: E11, E13, E14, O24.1, O24.2, O24.3<br>ICD-9: 250*A, 250*C, 250*X<br>ICD-8:250*	From the NPR data ICD-codes E11, E13, E14, 250*A, 250*C, 250*X and 250* that were recoded before child's date of birth were used in the definition<br><br>ICD-8 codes were used to define the date of first diagnosis, but subsequent ICD-9 codes were used to define the diabetes type [a]
	Gestational diabetes	MBR	Pre-defined variables, ICD-10: O24.0, O24.9	Pre-defined variables available since 2004
		NPR	ICD-10: O24.4, O24.9<br>ICD-9: 6480A, 6488A	
Norway	Any pre-pregnancy diabetes	MBR	Pre-defined variable	available before 1999
	Type 1 diabetes	MBR	Pre-defined variable	available since 1999
	Type 2 or other pre-pregnancy diabetes	MBR	Pre-defined variables	available since 1999
	Gestational diabetes	MBR	Pre-defined variable	
Sweden	Type 1 diabetes	NPR	E10	Diagnosis code recoded any time since 1997 was used in the definition for all children in the data [b]
	Pre-pregnancy diabetes	MBR	Pre-defined variable	In years 1990-1993, this variable may also have included previous gestational diabetes
**Mother's hypertensive disorders during pregnancy**				

Finland	Chronic hypertension or gestational hypertension (without pre-eclampsia or eclampsia in the same pregnancy)	NPR<br>MBR	ICD-10: O10 and O13<br>ICD-9/8: 6420X, 6421X, 6422X, 6423A, 6423B, 6423C, 63701	Diagnosis codes recoded during index pregnancy were used in the definition
	Pre-eclampsia or eclampsia	NPR<br>MBR	ICD-10: O11, O14, O15<br>ICD-9/8: 6427A, 6427B, 6427C, 6424A, 6424B, 6424C, 6425A, 6425B, 6425C, 63703, 63704, 63709, 6426A, 6426B, 6426C, 63710	Diagnosis codes recoded during index pregnancy were used in the definition
Norway	Chronic hypertension or gestational hypertension (without pre-eclampsia or eclampsia in the same pregnancy)	MBR	Pre-defined variables	
	Pre-eclampsia or eclampsia	MBR	Pre-defined variables	
Sweden	Chronic hypertension	MBR	Pre-defined variable	

Abbreviations: NPR, National Patient Register; MBR, Medical Birth Register

[a] In Finland, diabetes types were distinguishable in the ICD-codes from version 9 onwards (adopted in 1987)

[b] In Sweden, diabetes types were distinguishable in the ICD-codes from version 10 onwards (adopted in 1997)

ESM Table 2 Full characteristics of the study population by gestational age categories

Characteristic	Gestational age category (weeks)													
	Extremely preterm (23–27 weeks)		Very preterm (28–31 weeks)		Moderately preterm (32–33 weeks)		Late preterm (34–36 weeks)		Early term (37–38 weeks)		Full-term (39–41 weeks)		Post-term (42–45 weeks)	
	n	%	*n*	%	*n*	%	*n*	%	*n*	%	*n*	%	*n*	%
**Finland**														
Age, mother (years), mean (SD)	30.2	(5.7)	30.1	(5.7)	29.9	(5.7)	29.7	(5.6)	29.7	(5.4)	29.2	(5.2)	29.0	(5.2)
Parity, mother														
0	1650	49.4	4197	52.1	5875	53.8	32,174	48.8	113,803	38.5	477,304	39.9	40,023	54.6
1	857	25.6	2083	25.8	2784	25.5	18,454	28.0	98,327	33.2	419,392	35.1	19,840	27.0
2 or more	836	25.0	1782	22.1	2253	20.6	15,328	23.2	83,575	28.3	299,363	25.0	13,496	18.4
Education level, mother														
Low	603	18.0	1344	16.7	1738	15.9	10,275	15.6	43,825	14.8	163,033	13.6	10,541	13.6
Intermediate	1953	58.4	4811	59.7	6405	58.7	39,392	59.7	178,906	60.5	726,123	60.7	44,044	60.0
High	787	23.5	1907	23.7	2769	25.4	16,289	24.7	72,974	24.7	306,903	25.7	18,774	25.6
Diabetes, mother	199	6.0	769	9.5	1207	11.1	8469	12.8	36,297	12.3	92,623	7.7	4337	5.9
Hypertensive disorders, mother	525	15.7	2154	26.7	2524	23.1	11,527	17.5	31,994	10.8	68,202	5.7	2927	4.0
Sex														
Male	1815	54.3	4466	55.4	6096	55.9	35,953	54.5	156,745	53.0	601,976	50.3	37,700	51.4
Female	1528	45.7	3596	44.6	4816	44.1	30,003	45.5	138,960	47.0	594,083	49.7	35,659	48.6
Birth year														
1987–1989	358	10.7	782	9.7	960	8.8	6809	10.3	32,147	10.9	131,269	11.0	7248	9.9
1990–1999	1150	34.4	2809	34.8	3843	35.2	23,169	35.1	105,549	35.7	427,007	35.7	26,404	36.0
2000–2009	1105	33.0	2768	34.3	3746	34.3	21,333	32.3	95,039	32.1	379,577	31.7	25,041	34.1
2010–2016	730	21.8	1703	21.1	2363	21.7	14,645	22.2	62,970	21.3	258,206	22.6	14,666	20.0
Birthweight z score [a]														
less than -2	421	12.6	1264	15.7	1399	12.8	5473	8.3	12,286	4.1	24,009	2.0	1204	1.6
-2 to less than -1	418	12.5	1282	15.9	1691	15.5	11,190	17.0	46,925	15.9	168,332	14.1	9809	13.4
-1 to less than 0	785	23.5	1902	23.6	3090	28.3	20,669	31.3	100,597	34.0	438,499	36.7	27,154	37.0
0 to less than 1	937	28.0	2014	25.0	2845	26.1	17,785	27.0	87,322	29.5	392,644	32.8	24,713	33.7
1 to less than 2	555	16.6	1131	14.0	1342	12.3	7462	11.3	36,387	12.3	142,435	11.9	8869	12.1
2 or more	227	6.8	469	5.8	545	5.0	3377	5.1	12,188	4.1	30,140	2.5	1610	2.2
Age, father (years), mean (SD)	33.0	(6.4)	33.0	(6.4)	32.6	(6.4)	32.5	(6.3)	32.5	(6.1)	32.1	(6.0)	31.9	(6.0)
Education level, father														
Low	606	18.1	1665	20.7	2157	19.8	12,957	19.6	55,683	18.8	217,771	18.2	13,553	18.5
Intermediate	1752	52.4	4524	56.1	6234	57.1	38,136	57.8	173,012	58.5	702,672	58.8	42,921	58.5
High	648	19.4	1590	19.7	2250	20.6	13,542	20.5	62,718	21.2	259,837	21.7	15,573	21.2
Missing information	337	10.1	283	3.5	271	2.5	1321	2.0	4292	1.5	15,779	1.3	1312	1.8

Type 1 diabetes, father	6	0.2	37	0.4	52	0.4	240	0.4	1110	0.4	4528	0.4	267	0.4
Country of birth, father														
Finland	2900	86.7	7508	93.1	10,293	94.3	62,419	94.6	28,1813	95.3	1,139,806	95.3	69,314	94.5
Other high-income country	64	1.9	156	1.9	203	1.9	1313	2.0	5828	2.0	24,970	2.1	1656	2.3
Other country	47	1.4	131	1.6	164	1.5	1011	1.5	4200	1.4	17,372	1.4	1190	1.6
Missing information	332	9.9	267	3.3	252	2.3	1213	1.8	3864	1.3	13911	1.2	1199	1.6
**Norway**														
Age, mother (years), mean (SD)	30.1	(5.5)	29.7	(5.5)	29.7	(5.4)	29.5	(5.4)	29.8	(5.3)	29.4	(5.0)	28.7	(5.0)
Parity, mother														
0	1274	51.4	4430	55.9	5868	53.9	29,766	49.6	85,542	39.9	381,784	41.3	58,955	50.0
1	665	26.8	2019	25.5	2997	27.5	18,357	30.6	76,863	35.9	346,497	37.5	37,737	32.0
2 or more	541	21.8	1477	18.6	2029	18.6	11,939	19.9	51,866	24.2	195,935	21.2	21,148	18.0
Education level, mother														
Low	625	25.2	2016	25.4	2694	24.7	14,599	24.3	47,870	22.3	191,911	20.8	28,727	24.4
Intermediate	913	36.8	2988	37.7	4123	37.8	22,522	37.5	79,631	37.2	340,555	36.8	46,901	39.8
High	942	38.0	2992	36.9	4076	37.4	22,941	38.2	86,770	40.5	391,741	42.4	42,210	35.8
Diabetes, mother	51	2.1	247	3.1	373	3.4	2231	3.7	6990	3.3	11,723	1.3	489	0.4
Hypertensive disorders, mother	456	18.4	2014	25.4	2316	21.3	9507	15.8	17,964	8.4	39,441	4.3	4007	3.4
Sex														
Male	1290	52.0	4337	54.7	5929	54.4	32,173	53.6	110,484	51.6	469,223	50.8	63,016	53.5
Female	1190	48.0	3589	45.3	4965	45.6	27,889	46.3	103,787	48.4	454,993	49.2	54,779	46.5
Birth year														
1987–1989	121	4.9	529	6.7	840	7.7	4821	8.0	15,722	7.3	81,996	8.9	15,805	13.4
1990–1999	669	27.0	2472	31.2	3426	31.4	19,215	32.0	63,130	29.5	302,623	32.7	56,020	47.5
2000–2009	1022	41.2	3149	39.7	4247	39.0	22,480	37.4	83,089	38.8	319,200	34.5	33,387	28.3
2010–2016	668	26.9	1776	22.4	2381	21.9	13,546	22.5	52,330	24.4	220,397	23.8	12,628	10.7
Birthweight z score [a]														
less than -2	301	12.1	1203	15.2	1336	12.3	4894	8.1	9618	4.5	21,532	2.3	3708	3.5
-2 to less than -1	328	13.2	1352	17.1	1714	15.7	9921	16.5	34,820	16.2	132,397	14.3	18,494	15.7
-1 to less than 0	602	24.3	1092	24.0	3087	28.3	18,480	30.8	73,649	34.7	333,054	36.0	41,480	35.2
0 to less than 1	684	27.6	2055	35.9	2780	25.5	15,367	26.0	62,377	29.1	298,475	32.3	36,485	31.0
1 to less than 2	405	16.3	995	12.5	1297	11.9	7105	11.8	25,026	11.7	112,559	12.2	14,343	12.2
2 or more	160	6.4	419	5.3	680	6.2	4025	6.7	8781	4.1	26,199	2.8	3330	2.8
Age, father (years), mean (SD)	32.7	(6.2)	32.3	(6.4)	32.3	(6.2)	32.1	(6.1)	32.3	(6.0)	32.0	(5.8)	31.5	(5.8)
Education level, father														
Low	595	24.0	1885	23.8	2544	23.8	13,412	22.3	44,841	20.9	184,631	20.0	26,699	22.6
Intermediate	1090	43.9	3673	46.3	4937	45.3	27,822	46.3	98,721	46.1	422,980	45.8	54,768	46.5
High	718	28.9	2163	27.3	3142	28.8	17,493	29.1	66,314	31.0	299,988	32.4	34,232	29.0
Missing information	77	3.1	205	2.6	271	2.5	1335	2.2	4395	2.0	16717	1.8	2171	1.8
**Sweden**														
Age, mother (years), mean (SD)	30.6	(5.6)	30.4	(5.4)	30.2	(5.4)	30.1	(5.4)	30.4	(5.3)	30.0	(5.0)	30.2	(5.0)

Parity, mother														
0	2533	50.2	6856	49.3	9307	49.4	51,961	48.4	185,134	39.7	734,363	42.6	93,952	53.5
1	1512	30.0	4219	30.3	5815	30.9	32,705	30.5	173,350	37.2	659,824	38.3	53,409	30.4
2 or more	997	19.8	2827	20.3	3717	19.7	22,684	21.2	107,882	23.1	328,818	19.1	28,326	16.2
Education level, mother														
Low	675	13.4	1903	13.7	2493	13.2	13,937	13.0	57,586	12.4	187,287	10.9	18,392	10.5
Intermediate	2460	48.8	6923	49.8	9541	50.6	53,722	50.0	230,735	49.5	840,421	48.8	84,673	48.2
High	1907	37.8	5076	36.5	6805	36.1	39,691	37.0	178,045	38.2	695,297	40.4	72,622	41.3
Diabetes, mother	96	1.9	361	2.6	445	2.4	2719	2.5	8573	1.8	11,833	0.7	706	0.4
Hypertensive disorders, mother	61	1.2	198	1.4	188	1.0	842	0.8	2531	0.5	5876	0.3	468	0.3
Sex														
Male	2749	54.5	7578	54.5	10272	54.5	57,475	53.5	236,277	50.7	875,089	50.8	100,661	57.3
Female	2293	45.5	6324	45.5	8567	45.5	49,875	46.5	230,089	49.0	847,916	49.2	75,026	42.7
Birth year														
1987–1989	267	5.3	1331	9.6	1972	10.5	12,297	11.5	52,060	11.2	186,725	10.8	18,644	10.6
1990–1999	1371	27.2	4755	34.2	6542	34.7	37,107	34.6	156,383	33.5	598,321	34.7	61,232	34.9
2000–2009	1940	38.5	4651	33.4	6050	32.1	34,225	31.9	151,300	32.4	535,232	31.1	56,578	32.3
2010–2016	1464	29.0	3165	22.7	4275	22.7	23,721	22.1	106,623	22.9	402,727	23.4	39,233	22.3
Birthweight z score [a]														
less than -2	377	7.5	1380	9.9	1468	7.8	5786	5.4	13,023	2.8	26,709	1.6	2470	1.4
-2 to less than -1	490	9.7	1537	11.1	2103	11.2	12,388	11.5	53,695	11.5	193,074	11.2	19,196	10.9
-1 to less than 0	843	16.7	2581	18.6	4048	21.5	29,099	27.1	144,590	31.0	572,252	33.2	56,886	32.4
0 to less than 1	1336	26.5	5594	25.9	5533	29.4	33,703	31.4	155,104	33.3	602,012	34.9	61,719	35.1
1 to less than 2	1156	22.9	2936	21.1	3889	20.1	18,364	17.1	72,705	15.6	259,665	15.1	28,001	15.9
2 or more	840	16.7	1874	13.5	1908	10.1	8010	7.5	27,249	5.8	69,293	4.0	7415	4.2
Age, father (years), mean (SD)	32.9	(6.3)	32.8	(6.2)	32.6	(6.2)	32.5	(6.1)	32.8	(6.0)	32.5	(5.8)	32.7	(5.9)
Education level, father														
Low	768	15.2	2136	15.4	2844	15.1	15,723	14.7	66,571	14.3	230,047	13.4	22,671	12.9
Intermediate	2528	50.1	7300	52.5	9761	51.8	56,247	52.4	241,520	51.8	881,782	51.2	89,140	50.7
High	1527	30.3	4091	29.4	5733	30.4	32,589	30.4	147,579	31.6	574,252	33.3	59,928	34.1
Missing information	219	4.3	375	2.7	501	2.7	2791	2.6	10,696	2.3	36,924	2.1	3948	2.3

[a] Birthweight z scores calculated according to Sankilampi [22]

ESM Table 3 Association between gestational age and risk of type 1 diabetes – country-specific and pooled unadjusted and adjusted estimates

Country	Gestational age category (weeks)	Type 1 diabetes, *n*	Total, *n*	Model<br>Country: *n*											
				Unadjusted<br>FI: 1,653,396<br>NO: 1,337,689<br>SE: 2,510,191		Model 1[a]<br>FI: 1,653,396<br>NO: 1,337,689<br>SE: 2,510,191		Model 2[b] (main model)<br>FI: 1,653,396<br>NO: 1,337,689<br>SE: 2,510,191		Model 3[c]<br>FI: 1,629,801<br>NO: 1,312,749<br>SE: 2,454,737		Model 4[d]<br>FI: 1,629,801		Model 5[e]<br>FI: 1,653,377<br>NO: 1,337,689<br>SE: 2,246,097	
				HR	95% CI	HR	95% CI	HR	95% CI	HR	95% CI	HR	95% CI	HR	95% CI
Finland	23–27	12	3343	0.73	0.42, 1.29	0.72	0.41, 1.27	0.70	0.40, 1.23	0.71	0.40, 1.25	0.71	0.40, 1.26	0.69	0.39, 1.22
	28–31	45	8062	0.84	0.63, 1.13	0.82	0.62, 1.11	0.79	0.59, 1.06	0.81	0.60, 1.10	0.80	0.60, 1.08	0.79	0.58, 1.05
	32–33	90	10,912	1.21	0.98, 1.49	1.18	0.96, 1.46	1.10	0.90, 1.36	1.11	0.90, 1.37	1.09	0.88, 1.35	1.10	0.89, 1.35
	34–36	557	65,956	1.21	1.11, 1.32	1.19	1.10, 1.30	1.10	1.01, 1.20	1.10	1.01, 1.20	1.10	1.01, 1.20	1.10	1.00, 1.20
	37–38	2587	295,705	1.23	1.17, 1.28	1.22	1.17, 1.27	1.16	1.11, 1.22	1.16	1.11, 1.22	1.16	1.11, 1.21	1.16	1.11, 1.22
	39–41	8533	1,196,059	1.00	[Reference]	1.00	[Reference]	1.00	[Reference]	1.00	[Reference]	1.00	[Reference]	1.00	[Reference]
	42–45	502	73,359	0.96	0.87, 1.04	0.95	0.87, 1.04	0.96	0.87, 1.05	0.95	0.87, 1.04	0.95	0.87, 1.04	0.96	0.87, 1.05
Norway	23–27	6	2480	0.54	0.24, 1.21	0.58	0.26, 1.28	0.56	0.25, 1.26	0.58	0.26, 1.29	[f]		0.55	0.25, 1.23
	28–31	24	7926	0.65	0.44, 0.98	0.66	0.44, 0.98	0.63	0.42, 0.95	0.59	0.39, 0.90			0.61	0.41, 0.92
	32–33	55	10,894	1.09	0.83, 1.42	1.09	0.83, 1.42	1.04	0.80, 1.36	1.06	0.81, 1.39			1.02	0.78, 1.33
	34–36	320	60,062	1.15	1.03, 1.29	1.15	1.02, 1.29	1.09	0.97, 1.22	1.19	0.97, 1.22			1.07	0.96, 1.21
	37–38	1160	214,271	1.18	1.11, 1.26	1.21	1.13, 1.29	1.16	1.09, 1.24	1.17	1.09, 1.25			1.15	1.08, 1.23
	39–41	4253	924,216	1.00	[Reference]	1.00	[Reference]	1.00	[Reference]	1.00	[Reference]			1.00	[Reference]
	42–45	546	117,840	0.95	0.87, 1.04	0.84	0.77, 0.92	0.85	0.78, 0.93	0.86	0.78, 0.94			0.85	0.78, 0.93
Sweden	23–27	17	5042	0.65	0.40, 1.04	0.65	0.41, 1.05	0.61	0.38, 0.98	0.63	0.39, 1.01	[f]		0.62	0.38, 1.00
	28–31	79	13,902	0.89	0.71, 1.11	0.88	0.71, 1.10	0.83	0.66, 1.03	0.79	0.63, 0.99			0.79	0.62, 0.99
	32–33	123	18,839	1.01	0.84, 1.20	1.00	0.84, 1.19	0.94	0.79, 1.12	0.94	0.79, 1.13			0.91	0.75, 1.10
	34–36	865	107,350	1.23	1.15, 1.32	1.22	1.14, 1.31	1.15	1.07, 1.23	1.16	1.08, 1.24			1.13	1.05, 1.21
	37–38	3558	466,366	1.18	1.13, 1.22	1.18	1.13, 1.22	1.13	1.09, 1.18	1.13	1.09, 1.18			1.11	1.07, 1.16
	39–41	11,189	1,723,005	1.00	[Reference]	1.00	[Reference]	1.00	[Reference]	1.00	[Reference]			1.00	[Reference]
	42–45	1025	175,687	0.89	0.84, 0.95	0.88	0.83, 0.94	0.89	0.83, 0.95	0.89	0.83, 0.95			0.87	0.81, 0.94
Pooled	23–27	35	10,865	0.66	0.47, 0.91	0.66	0.47, 0.92	0.63	0.45, 0.88	0.65	0.46, 0.90			0.63	0.45, 0.88
	28–31	148	29,890	0.83	0.71, 0.98	0.83	0.70, 0.97	0.78	0.67, 0.92	0.76	0.64, 0.90			0.76	0.64, 0.89
	32–33	268	40,645	1.09	0.97, 1.23	1.07	0.95, 1.21	1.01	0.90, 1.14	1.02	0.90, 1.15			1.00	0.88, 1.13
	34–36	1742	233,368	1.21	1.15, 1.27	1.20	1.14, 1.26	1.12	1.07, 1.18	1.16	1.08, 1.24			1.11	1.06, 1.17
	37–38	7305	976,342	1.20	1.17, 1.23	1.20	1.17, 1.23	1.15	1.11, 1.18	1.15	1.11, 1.18			1.13	1.10, 1.17
	39–41	23,964	3,843,280	1.00	[Reference]	1.00	[Reference]	1.00	[Reference]	1.00	[Reference]			1.00	[Reference]
	42–45	2073	366,886	0.92	0.88, 0.96	0.89	0.85, 0.93	0.90	0.85, 0.94	0.90	0.86, 0.94			0.89	0.85, 0.93

FI, Finland; NO, Norway; SE, Sweden

[a] Model 1: adjusted for child's sex and birth year
[b] Model 2: adjusted for child's sex, birth year and birthweight z score and mother's age, education level, parity, diabetes during pregnancy and hypertensive disorders during pregnancy
[c] Model 3: adjusted for child's sex, birth year and birthweight z score and mother's age, education level, parity, diabetes during pregnancy and hypertensive disorders during pregnancy as well as father's age and education
[d] Model 4: adjusted for child's sex, birth year and birthweight z score and mother's age, education level, parity, diabetes during pregnancy, hypertensive disorders during pregnancy, father's age and education as well as father's type 1 diabetes and country of birth
[e] Model 5: adjusted for child's sex, birth year and birthweight z score and mother's age, education level, parity, diabetes during pregnancy, and hypertensive disorders during pregnancy as well as caesarean section
[f] Information on father's type 1 diabetes and country of birth was not available in Norway and Sweden

ESM Table 4. Corrected p-values of the main analyses according to Holm procedure[1]

**Comparison**	**p-value from original, pooled analysis**	**Re-ordering of the p-values**	**α/k**<br>**α/(k-1)**
H1: 23–27 wk vs. 39–41 wk	0.006	H6: <0.001	0.05/6 = 0.008
H2: 28–31 wk vs. 39–41 wk	0.003	H5: <0.001	0.05/5 = 0.010
H3: 32–33 wk vs. 39–41 wk	0.859	H4: <0.001	0.05/4 = 0.013
H4: 34–36 wk vs. 39–41 wk	<0.001	H2: 0.003	0.05/3 = 0.017
H5: 37–38 wk vs. 39–41 wk	<0.001	H1: 0.006	0.05/2 = 0.025
H6: 42–45 wk vs. 39–41 wk	<0.001	H3: 0.859	0.05/1 = 0.05

[1] Holm S. A Simple Sequentially Rejective Multiple Test Procedure. Scandinavian Journal of Statistics 1979;6:65–70.

ESM Table 5 Country-specific associations of combinations of gestational age and birthweight z score with the risk of type 1 diabetes

Country	Gestational age category (weeks)	**Birthweight z score category** [a] Country: type 1 diabetes/total, *n*											
		**< -2**		**-2 to < -1**		**-1 to < 0**		**0 to < +1**		**+1 to < +2**		**≥ +2**	
		FI: 245/46,056<br>NO: 186/42,592<br>SE: 316/51,213		FI: 1627/239,647<br>NO: 837/199,026<br>SE: 1713/282,483		FI: 4167/592,696<br>NO: 2081/472,254<br>SE: 5195/810,299		FI: 4102/528,260<br>NO: 2099/418,493<br>SE: 5919/836,001		FI: 1677/198,181<br>NO: 890/161,730<br>SE: 2772/386,606		FI: 508/48,556<br>NO: 271/43,594<br>SE: 941/116,589	
		HR[b]	95%CI	HR[b]	95%CI	HR[b]	95%CI	HR[b]	95%CI	HR[b]	95%CI	HR[b]	95%CI
Finland	<32	0.36	0.14, 0.97	0.74	0.38, 1.42	0.91	0.57, 1.47	0.84	0.52, 1.35	0.69	0.34, 1.37	0.37	0.09, 1.48
	32–33	0.65	0.31, 1.37	0.76	0.41, 1.42	1.21	0.84, 1.76	0.95	0.62, 1.47	1.40	0.86, 2.29	1.21	0.60, 2.42
	34–36	0.49	0.31, 0.75	1.02	0.82, 1.27	1.03	0.88, 1.21	1.12	0.95, 1.31	1.18	0.94, 1.47	1.23	0.93, 1.62
	37–38	0.95	0.77, 1.18	0.96	0.86, 1.08	1.08	1.00, 1.17	1.15	1.06, 1.24	1.33	1.19, 1.47	1.18	1.00, 1.39
	39–41	0.71	0.59, 0.85	0.93	0.87, 0.99	0.93	0.88, 0.97	1.00	[Reference]	1.00	0.93, 1.07	1.11	0.98, 1.25
	42–45	0.31	0.10, 0.97	0.92	0.72, 1.17	0.86	0.74, 1.00	0.97	0.84, 1.13	1.05	0.82, 1.33	0.89	0.49, 1.61
Norway	<32	0.68	0.28, 1.64	0.47	0.18, 1.26	0.41	0.17, 0.98	0.81	0.45, 1.47	0.42	0.13, 1.30	0.52	0.13, 2.09
	32–33	0.43	0.14, 1.34	0.34	0.11, 1.06	1.24	0.79, 1.96	1.07	0.65, 1.79	0.96	0.46, 2.03	1.67	0.83, 3.35
	34–36	1.10	0.76, 1.60	1.00	0.75, 1.33	0.94	0.76, 1.17	1.21	0.99, 1.50	0.80	0.57, 1.13	0.94	0.65, 1.36
	37–38	0.81	0.59, 1.11	0.94	0.79, 1.11	1.00	0.89, 1.13	1.16	1.03, 1.31	1.38	1.18, 1.61	1.22	0.96, 1.55
	39–41	0.81	0.66, 1.00	0.82	0.75, 0.91	0.89	0.83, 0.96	1.00	[Reference]	1.08	0.98, 1.18	1.02	0.86, 1.21
	42–45	0.59	0.36, 0.99	0.68	0.54, 0.85	0.78	0.67, 0.90	0.90	0.77, 1.05	0.79	0.61, 1.02	0.87	0.54, 1.44
Sweden	<32	0.60	0.29, 1.26	0.60	0.30, 1.20	1.11	0.75, 1.66	0.72	0.48, 1.09	0.56	0.34, 0.93	0.92	0.58, 1.46
	32–33	0.84	0.43, 1.62	0.61	0.32, 1.18	1.05	0.73, 1.51	0.91	0.65, 1.28	0.69	0.44, 1.09	1.47	0.98, 2.18
	34–36	0.69	0.48, 1.00	0.82	0.65, 1.03	1.05	0.92, 1.21	1.21	1.07, 1.37	1.33	1.15, 1.55	1.27	1.03, 1.57
	37–38	0.83	0.66, 1.04	0.96	0.86, 1.07	1.09	1.01, 1.16	1.19	1.12, 1.27	1.13	1.04, 1.23	1.14	1.00, 1.30
	39–41	0.94	0.81, 1.09	0.90	0.85, 0.96	0.94	0.90, 0.99	1.00	[Reference]	1.04	0.98, 1.10	1.05	0.95, 1.15
	42–45	1.04	0.67, 1.64	0.84	0.69, 1.01	0.85	0.76, 0.95	0.87	0.78, 0.97	0.92	0.79, 1.06	0.81	0.60, 1.11

FI, Finland; NO, Norway; SE, Sweden

[a] Birthweight z scores calculated according to Sankilampi et al [22]

[b] HRs are adjusted for child's sex and birth year and mother's age, parity, education level, diabetes during pregnancy, and hypertensive disorders during pregnancy (model 2)

ESM Table 6 Country-specific total number and number of individuals with type 1 diabetes by gestational age and birthweight z score categories

Country	Gestational age category (weeks)	Birthweight z score category [a] Country: type 1 diabetes/total, *n*					
		**< -2**	**-2 to < -1**	**-1 to < 0**	**0 to < +1**	**+1 to < +2**	**≥ +2**
		FI: 245/46,056<br>NO: 186/42,592<br>SE: 316/51,213	FI: 1627/239,647<br>NO: 837/199,026<br>SE: 1713/282,483	FI: 4167/592,696<br>NO: 2081/472,254<br>SE: 5195/810,299	FI: 4102/528,260<br>NO: 2099/418,493<br>SE: 5919/836,001	FI: 1677/198,181<br>NO: 890/161,730<br>SE: 2772/386,606	FI: 508/48,556<br>NO: 271/43,594<br>SE: 941/116,589
		Type 1 diabetes/ total, *n*	Type 1 diabetes/ total, *n*	Type 1 diabetes/ total, *n*	Type 1 diabetes/ total, *n*	Type 1 diabetes/ total, *n*	Type 1 diabetes/ total, *n*
Finland	<32	<5/1685	9/1700	17/2687	17/2951	8/1686	<5/696
	32–33	7/1399	10/1691	28/3090	21/2845	16/1342	8/545
	34–36	20/5473	84/11,190	160/20,699	158/17,785	80/7462	55/3377
	37–38	85/12286	332/46,925	820/100,597	787/87,322	405/36,387	158/12188
	39–41	126/24009	1125/168,332	2969/438,499	2941/392,644	1098/142,435	274/30140
	42–45	<5/1204	67/9809	173/27,154	178/24,713	70/8869	11/1610
Norway	<32	5/1504	<5/1680	5/2504	11/2739	<5/1400	<5/579
	32–33	<5/1336	<5/1714	19/3087	15/2780	7/1297	8/680
	34–36	28/4894	49/9921	84/18,480	96/15,637	33/7105	30/4025
	37–38	39/9618	156/34,820	351/73,649	354/62,377	186/25,026	74/8781
	39–41	96/21532	546/132,397	1433/333,054	1438/298,475	599/112,559	141/26199
	42–45	15/3708	79/18,494	189/41,480	185/36,485	62/14,343	16/3330
Sweden	<32	7/1757	8/2027	25/3424	23/4930	15/4092	18/2714
	32–33	9/1468	9/2103	29/4048	34/5533	19/3779	23/1908
	34–36	29/5786	71/12,388	211/29,099	281/33,703	180/18,364	93/8010
	37–38	76/13023	348/53,695	1047/144,590	1246/155,104	582/72,705	259/27249
	39–41	176/26709	1166/193,074	3556/572,252	3977/602,012	1806/259,665	508/69293
	42–45	19/2470	111/19,196	327/56,886	358/61,719	170/28,001	40/7415

FI, Finland; NO, Norway; SE, Sweden

[a] Birthweight z scores calculated according to Sankilampi et al [22]

ESM Table 7 Country-specific associations of combination of gestational age and mother's type 1 diabetes status with risk of type 1 diabetes in offspring

Country	Gestational age category (weeks)	Maternal type 1 diabetes[a]<br>Country: type 1 diabetes/total, *n*					
		**No**<br>FI: 12,044/1,646,018<br>NO: 2661/815,337<br>SE: 16,106/2,488,805			**Yes**<br>FI: 282/7378<br>NO: 86/3914<br>SE: 750/21,386		
		Type 1 diabetes/ total, *n*	HR[b]	95%CI	Type 1 diabetes/ total, *n*	HR[b]	95%CI
Finland	<32	50/11,199	0.73	0.56, 0.97	7/206	6.44	3.07, 13.53
	32–33	76/10,637	1.05	0.84, 1.32	14/275	8.14	4.81, 13.77
	34–36	499/64,167	1.12	1.02, 1.22	58/1789	5.40	4.16, 7.00
	37–38	2429/291,875	1.17	1.12, 1.22	158/3830	6.22	5.30, 7.29
	39–41	8489/1,194,796	1.00	[Reference]	44/1263	5.55	4.11, 7.25
	42–45	501/73,344	0.95	0.87, 1.05	<5/15	9.36	1.32, 66.47
Norway	<32	13/6970	0.58	0.34, 1.01	0/101	[c]	
	32–33	25/6925	1.11	0.74, 1.64	5/130	11.47	4.75, 27.67
	34–36	146/37,571	1.21	1.02, 1.44	15/678	6.59	3.95, 11.01
	37–38	537/141,577	1.20	1.09, 1.32	38/1745	7.02	5.07, 9.72
	39–41	1785/571,880	1.00	[Reference]	28/1249	6.72	4.62, 9.77
	42–45	155/50,414	0.83	0.70, 0.98	0/11	[c]	
Sweden	<32	83/18,517	0.75	0.61, 0.93	13/427	4.82	2.80, 8.31
	32–33	109/18,419	0.93	0.77, 1.12	14/420	5.04	2.98, 8.51
	34–36	772/104,842	1.15	1.06, 1.23	93/2508	5.78	4.71, 7.10
	37–38	3294/458,726	1.14	1.09, 1.18	264/7640	5.38	4.75, 6.09
	39–41	10841/1,713,160	1.00	[Reference]	348/9845	4.93	4.44, 5.50
	42–45	1007/175,141	0.89	0.83, 0.95	18/546	4.15	2.61, 6.59

FI, Finland; NO, Norway; SE, Sweden

[a] In Norway, analyses were restricted to those born in 1999 or later, as data on maternal pre-pregnancy diabetes subtypes are available only from 1999

[b] HR are adjusted for child's sex, birth year and birthweight z score and mother's age, parity, education level, and hypertensive disorders during pregnancy (model 2 without diabetes during pregnancy)

[c] Not enough individuals for the analysis

ESM Table 8 Country-specific associations of combination of gestational age and maternal hypertensive disorders during pregnancy with risk of type 1 diabetes in offspring

**Country**	**Gestational age category (weeks)**	**Maternal hypertensive disorder during pregnancy** Country: type 1 diabetes/total, *n*					
		**No** FI: 11,379/1,533,543 NO: 5994/1,261,984 SE: 16,779/2,500,027			**Yes** FI: 947/119,853 NO: 370/75,705 SE: 77/10,164		
		Type 1 diabetes/ total, *n*	HR[a]	95% CI	Type 1 diabetes/ total, *n*	HR[a]	95% CI
Finland	<32	47/8726	0.83	0.62, 1.10	10/2679	0.61	0.33, 1.14
	32–33	70/8388	1.13	0.89, 1.43	20/2524	1.07	0.69, 1.67
	34–36	455/54,429	1.10	1.00, 1.21	102/11,527	1.17	0.96, 1.42
	37–38	2302/263,711	1.17	1.11, 1.22	285/31,994	1.22	1.08, 1.38
	39–41	8022/1,127,857	1.00	[Reference]	511/68,202	1.09	1.00, 1.20
	42–45	483/70,432	0.96	0.88, 1.05	19/2927	0.95	0.60, 1.48
Norway	<32	21/7,936	0.55	0.36, 0.85	9/2470	0.83	0.43, 1.61
	32–33	44/8,578	1.05	0.78, 1.41	11/2316	0.99	0.55, 1.80
	34–36	272/50,555	1.10	0.97, 1.24	48/9507	0.99	0.74, 1.32
	37–38	1068/196,307	1.17	1.10, 1.26	92/17,964	0.99	0.86, 1.31
	39–41	4061/884,775	1.00	[Reference]	192/39,441	1.06	0.92, 1.22
	42–45	528/113,833	0.85	0.78, 0.94	18/4007	0.82	0.52, 1.31
Sweden	<32	95/18,685	0.79	0.64, 0.96	<5/259	0.47	0.07, 3.37
	32–33	122/18,651	0.95	0.80, 1.13	<5/188	0.59	0.08, 4.19
	34–36	860/106,508	1.16	1.08, 1.24	5/842	0.69	0.29, 1.67
	37–38	3541/463,835	1.14	1.09, 1.18	17/2531	0.95	0.59, 1.52
	39–41	11139/1,717,129	1.00	[Reference]	50/5876	1.27	0.96, 1.68
	42–45	1022/175,219	0.89	0.83, 0.95	<5/468	0.92	0.30, 2.84

FI, Finland; NO, Norway; SE, Sweden

[a] HR are adjusted for child's sex, birth year and birthweight z score and mother's age, parity, education level, and diabetes during pregnancy (model 2 without hypertensive disorders during pregnancy)

ESM Table 9. Cause-specific hazards of type 1 diabetes and death

**Country**	**Gestational age category (weeks)**	**Total**	**Type 1 diabetes**			**Death**			**Cause-specific hazards**			
									**Type 1 diabetes**		**Death (competing event)**	
		n	*n*	%	Age at diagnosis (years), median	*n*	%	Age at death (years), median	HR[b]	95% CI	HR[b]	95% CI
Finland	23–27	3343	12	0.4	8.6	915	27.4	<0.1	0.70	0.40, 1.23	79.64	74.06, 85.65
	28–31	8062	45	0.6	7.1	492	6.1	<0.1	0.79	0.59, 1.06	12.82	11.65, 14.11
	32–33	10,912	90	0.8	7.2	256	2.4	<0.1	1.10	0.90, 1.36	4.89	4.30, 5.55
	34–36	65,956	557	0.8	7.9	810	1.2	0.3	1.10	1.01, 1.20	2.56	2.38, 2.76
	37–38	295,705	2587	0.9	7.9	1745	0.6	2.9	1.16	1.11, 1.22	1.28	1.21, 1.35
	39–41	1,196,059	8533	0.7	8.2	5227	0.4	8.3	1.00	[Reference]	1.00	[Reference]
	42–45	73,359	502	0.7	8.8	332	0.5	7.9	0.96	0.87, 1.05	1.07	0.96, 1.20
Norway	23–27	2480	6	0.2	14.1	98	4.0	<0.1	0.56	0.25, 1.26	25.02	20.34, 30.77
	28–31	7926	24	0.3	13.5	43	0.5	0.2	0.63	0.42, 0.95	2.84	2.09, 3.86
	32–33	10,894	55	0.5	11.7	57	0.5	3.8	1.04	0.80, 1.36	2.70	2.07, 3.53
	34–36	60,062	320	0.5	12.3	159	0.3	11.1	1.09	0.97, 1.22	1.39	1.18, 1.65
	37–38	214,271	1160	0.5	12.3	418	0.2	17.9	1.16	1.09, 1.24	1.10	0.98, 1.22
	39–41	924,216	4253	0.5	13.1	1648	0.2	19.3	1.00	[Reference]	1.00	[Reference]
	42–45	117,840	546	0.5	14.7	280	0.2	20.5	0.85	0.78, 0.93	1.08	0.95, 1.23
Sweden	23–27	5042	17	0.3	11.8	660	13.1	<0.1	0.61	0.38, 0.98	51.75	47.61, 56.26
	28–31	13,902	79	0.6	12.2	332	2.4	<0.1	0.83	0.66, 1.03	7.20	6.43, 8.06
	32–33	18,839	123	0.7	10.6	234	1.2	0.2	0.94	0.79, 1.12	3.79	3.32, 4.32
	34–36	107,350	865	0.8	10.3	688	0.6	1.1	1.15	1.07, 1.23	2.00	1.84, 2.17
	37–38	466,366	3558	0.8	10.6	1726	0.4	11.1	1.13	1.09, 1.18	1.23	1.17, 1.30
	39–41	1,723,005	11,189	0.7	10.8	4957	0.3	16.2	1.00	[Reference]	1.00	[Reference]
	42–45	175,687	1025	0.6	10.6	549	0.3	14.7	0.89	0.83, 0.95	1.09	0.99, 1.19

[a]In Norway and Sweden, those who died before the start of the follow-up (year 2008 in Norway and 1997 in Sweden) were excluded from the study population

[b] HR are adjusted for child's sex and birth year and mother's age, education level, parity, diabetes during pregnancy and hypertensive disorders during pregnancy (model 2).

ESM Table 10. Association between gestational age and the risk of type 1 diabetes – country-specific and pooled adjusted estimates from analyses investigating death as potential source of informative censoring with different assumptions

Country	Gestational age category (weeks)	Type 1 diabetes among those who did not die		Magnitude of cumulative incidence of type 1 diabetes diagnosis assigned to those who died[a]: 0.2 times lower than incidence among those who did not die		Equal to incidence among those who did not die		1.5 times higher than incidence among those who did not die		2.0 times higher than incidence among those who did not die		2.5 times higher than incidence among those who did not die	
		n	%	HR[b]	95% CI	HR[b]	95% CI	HR[b]	95% CI	HR[b]	95% CI	HR[b]	95% CI
Finland	23–27	12	0.5	1.04	0.66, 1.65	1.21	0.79, 1.86	1.33	0.88, 2.00	1.55	1.06, 2.27	1.66	1.15, 2.40
	28–31	45	0.6	0.86	0.65, 1.14	0.86	0.65, 1.14	0.93	0.71, 1.22	0.96	0.73, 1.25	1.01	0.78, 1.31
	32–33	90	0.8	1.11	0.90, 1.37	1.12	0.91, 1.38	1.12	0.91, 1.38	1.13	0.92, 1.39	1.13	0.92, 1.39
	34–36	557	0.9	1.10	1.01, 1.20	1.10	1.01, 1.21	1.10	1.01, 1.20	1.11	1.02, 1.21	1.12	1.03, 1.22
	37–38	2587	0.9	1.16	1.11, 1.22	1.16	1.11, 1.22	1.16	1.11, 1.22	1.16	1.11, 1.21	1.16	1.11, 1.22
	39–41	8533	0.7	1.00	[Reference]	1.00	[Reference]	1.00	[Reference]	1.00	[Reference]	1.00	[Reference]
	42–45	502	0.7	0.96	0.87, 1.05	0.96	0.87, 1.05	0.96	0.87, 1.05	0.96	0.87, 1.04	0.95	0.87, 1.04
	Total	12,326	0.8										
Norway	23–27	6	0.3	0.63	0.30, 1.32	0.63	0.30, 1.32	0.63	0.30, 1.32	0.63	0.30, 1.32	0.72	0.36, 1.44
	28–31	24	0.3	0.61	0.41, 0.92	0.61	0.41, 0.92	0.61	0.41, 0.92	0.61	0.41, 0.92	0.61	0.41, 0.92
	32–33	55	0.5	1.03	0.78, 1.34	1.03	0.78, 1.34	1.02	0.78, 1.34	1.02	0.78, 1.34	1.02	0.78, 1.34
	34–36	320	0.5	1.08	0.96, 1.21	1.08	0.96, 1.21	1.08	0.96, 1.21	1.08	0.96, 1.21	1.08	0.96, 1.21
	37–38	1160	0.5	1.15	1.08, 1.23	1.15	1.08, 1.23	1.15	1.08, 1.23	1.15	1.08, 1.23	1.15	1.08, 1.23
	39–41	4253	0.5	1.00	[Reference]	1.00	[Reference]	1.00	[Reference]	1.00	[Reference]	1.00	[Reference]
	42–45	546	0.5	0.87	0.79, 0.95	0.87	0.79, 0.95	0.87	0.79, 0.95	0.87	0.79, 0.95	0.87	0.79, 0.95
	Total	6364	0.5										
Sweden	23–27	17	0.4	0.76	0.49, 1.16	0.76	0.49, 1.16	0.79	0.52, 1.20	0.93	0.63, 1.37	0.93	0.63, 1.37
	28–31	79	0.6	0.86	0.69, 1.07	0.86	0.69, 1.07	0.86	0.69, 1.07	0.88	0.71, 1.09	0.88	0.71, 1.09
	32–33	123	0.7	0.95	0.80, 1.13	0.95	0.80, 1.13	0.96	0.80, 1.14	0.96	0.81, 1.15	0.96	0.81, 1.15
	34–36	865	0.8	1.16	1.08, 1.24	1.16	1.08, 1.24	1.16	1.08, 1.24	1.16	1.08, 1.24	1.16	1.08, 1.24
	37–38	3558	0.8	1.14	1.09, 1.18	1.14	1.09, 1.18	1.14	1.09, 1.18	1.13	1.09, 1.18	1.13	1.09, 1.18
	39–41	11,189	0.7	1.00	[Reference]	1.00	[Reference]	1.00	[Reference]	1.00	[Reference]	1.00	[Reference]
	42–45	1025	0.6	0.89	0.83, 0.95	0.89	0.83, 0.95	0.89	0.83, 0.95	0.89	0.84, 0.95	0.89	0.84, 0.95
	Total	16,856	0.7										
Pooled	23–27	35	0.3	0.84	0.63, 1.12	0.90	0.68, 1.20	0.96	0.73, 1.27	1.12	0.87, 1.44	1.17	0.92, 1.51
	28–31	148	0.5	0.82	0.70, 0.96	0.82	0.70, 0.96	0.84	0.72, 0.98	0.86	0.74, 1.00	0.88	0.75, 1.02
	32–33	268	0.7	1.02	0.90, 1.14	1.02	0.90, 1.15	1.02	0.91, 1.16	1.03	0.91, 1.16	1.03	0.91, 1.16
	34–36	1742	0.7	1.13	1.07, 1.18	1.13	1.07, 1.18	1.13	1.07, 1.18	1.13	1.08, 1.19	1.13	1.08, 1.19
	37–38	7305	0.7	1.15	1.12, 1.18	1.15	1.12, 1.18	1.15	1.12, 1.18	1.15	1.12, 1.18	1.14	1.11, 1.18
	39–41	23,964	0.6	1.00	[Reference]	1.00	[Reference]	1.00	[Reference]	1.00	[Reference]	1.00	[Reference]
	42–45	2073	0.6	0.90	0.86, 0.95	0.90	0.86, 0.95	0.90	0.86, 0.95	0.90	0.86, 0.94	0.90	0.86, 0.94
	Total	35,535	0.6										

[a] Artificial type 1 diabetes diagnoses are randomly assigned to those who died during the study period [b] HR are adjusted for child's sex and birth year and mother's age, education level, parity, diabetes during pregnancy and hypertensive disorders during pregnancy (model 2).

ESM Table 11 Association between gestational age and the risk of type 1 diabetes. Comparison of the results from the main and sensitivity analysis of different time periods according to the start of the availability of type 1 diabetes diagnosis data in Sweden (since 1997) and in Norway (since 2008)

**Country**	**Birth years**														
**Gestational age category (weeks)**	**1987–2016 (main analysis)**					**1997–2016**					**2008–2016**				
	Type 1 diabetes/ total, *n*	Follow-up time (years), md	Age at diagnosis (years), md	HR[a]	95% CI	Type 1 diabetes/ total, *n*	Follow-up time (years), md	Age at diagnosis (years), md	HR[a]	95% CI	Type 1 diabetes/ total, *n*	Follow-up time (years), md	Age at diagnosis (years), md	HR[a]	95% CI
**Finland**															
<32	57/11,405	12.8	7.4	0.77	0.59, 1.00	26/7438	9.1	6.6	0.63	0.43, 0.93	6/3220	4.4	4.0	0.80	0.36, 1.80
32–33	90/10,912	14.6	7.2	1.10	0.90, 1.36	53/7277	10.0	5.9	1.17	0.90, 1.54	12/3131	4.5	4.8	1.50	0.85, 2.68
34–36	557/65,956	15.3	7.9	1.10	1.01, 1.20	307/42,590	10.0	6.1	1.14	1.01, 1.29	52/18,868	4.5	3.3	1.06	0.80, 1.42
37–38	2587/295,705	15.7	7.9	1.16	1.11, 1.22	1357/187,449	10.1	5.9	1.18	1.11, 1.26	239/81,801	4.6	3.4	1.21	1.04, 1.40
39–41	8533/1,196,059	15.7	8.2	1.00	[Reference]	4316/754,212	9.9	6.4	1.00	[Reference]	761/336,843	4.7	3.4	1.00	[Reference]
42–45	502/73,359	15.4	8.8	0.96	0.87, 1.05	248/46,657	10.0	6.7	0.92	0.81, 1.04	42/20,150	5.1	3.2	0.87	0.64, 1.19
**Norway**															
<32	30/10,406	10.0	13.9	0.62	0.43, 0.89						<5/3269	5.5	4.0	0.43	0.11, 1.74
32–33	44/10,894	10.0	11.7	1.04	0.80, 1.36						6/3204	5.8	3.9	1.21	0.54, 2.73
34–36	320/60,062	10.0	12.3	1.09	0.97, 1.22						40/17979	5.7	5.1	1.48	1.06, 2.06
37–38	1160/214,271	10.0	12.3	1.16	1.09, 1.24						131/69,520	5.7	3.8	1.28	1.05, 1.57
39–41	4253/924,216	10.0	13.1	1.00	[Reference]						397/286,547	5.6	4.3	1.00	[Reference]
42–45	546/117,840	10.0	14.7	0.85	0.78, 0.93						19/18,315	6.6	5.6	0.65	0.41, 1.03
**Sweden**															
<32	96/18,944	14.2	12.1	0.78	0.64, 0.96	48/12,893	9.7	8.4	0.77	0.58, 1.02	10/6025	5.4	3.7	0.86	0.46, 1.61
32–33	123/18,839	15.7	10.6	0.94	0.79, 1.12	71/11,994	10.5	8.4	1.13	0.89, 1.44	12/5474	5.5	4.4	1.07	0.61, 1.90
34–36	865/107,350	16.0	10.6	1.15	1.07, 1.23	419/67,050	10.5	7.6	1.28	1.06, 1.30	75/30,708	5.6	3.8	1.16	0.92, 1.47
37–38	3558/466,366	15.6	10.3	1.13	1.09, 1.18	1729/295,975	10.5	7.6	1.15	1.09, 1.21	340/138,037	5.5	4.0	1.23	1.08, 1.39
39–41	11189/1,723,005	15.8	10.6	1.00	[Reference]	5222/1,082,747	10.3	7.7	1.00	[Reference]	967/516,482	5.5	3.9	1.00	[Reference]
42–45	1025/175,687	16.0	10.8	0.89	0.83, 0.95	476/11,1255	10.7	7.9	0.87	0.79, 0.95	81/50,581	5.4	3.5	0.85	0.68, 1.07
**Pooled**															
<32	183/40,755			0.75	0.65, 0.87						<20/12,514			0.78	0.49, 1.24
32–33	257/40,645			1.01	0.90, 1.14						30/11,809			1.25	0.87, 1.80
34–36	1742/233,368			1.12	1.07, 1.18						167/67,555			1.19	1.02, 1.40
37–38	7305/976,342			1.15	1.11, 1.18						710/215,737			1.23	1.13, 1.34
39–41	23964/3,843,280			1.00	[Reference]						2125/1,139,872			1.00	[Reference]
42–45	2073/366,886			0.90	0.85, 0.94						142/89,046			0.82	0.70, 0.98

Md, median

[a] HR are adjusted for child's sex, birth year and birthweight z score and mother's age, education level, parity, diabetes during pregnancy and hypertensive disorder during pregnancy (model 2)

ESM Table 12 Country-specific and pooled associations between gestational age and the risk of type 1 diabetes from the sibling analyses

Country	Gestational age category (weeks)	Type 1 diabetes/ total, *n*	HR[a]	95% CI	Number of individuals included and excluded from the sibling analyses
Finland	<32	41/7780	0.65	0.40, 1.05	Main analysis cohort *n*= 1,653,396
	32–33	70/8059	1.07	0.73, 1.56	No father's identification number n= 23,561 (type 1 diabetes *n*= 138)
	34–36	415/49,502	1.07	0.91, 1.25	No full siblings in the data *n*= 370,972 (type 1 diabetes *n*= 2784)
	37–38	1983/226,585	1.14	1.05, 1.24	Final full sibling cohort *n*= 1,258,863 (76%)
	39–41	6543/913,933	1.00	[Reference]	
	42–45	352/53,004	0.98	0.84, 1.05	
	Total	9404/1,258,863			
Norway	<32	16/7186	1.12	0.52, 2.41	Main analysis cohort *n*= 1,337,689
	32–33	41/8077	1.61	0.93, 2.78	No father's identification number *n*= 12,041 (type 1 diabetes *n*= 28)
	34–36	237/44,621	0.87	0.71, 1.07	No full siblings in the data n= 331,047 (type 1 diabetes *n*= 1628)
	37–38	864/160,699	1.05	0.94, 1.18	Final full sibling cohort *n*= 994,601 (74%)
	39–41	3176/690,384	1.00	[Reference]	
	42–45	374/83,634	0.88	0.75, 1.02	
	Total	4708/994,601			
Sweden	<32	71/13,508	0.88	0.60, 1.31	Main analysis cohort *n*= 2,510,191
	32–33	88/14,199	0.94	0.66, 1.33	No father's identification number *n*= 14,691 (type 1 diabetes *n*= 49)
	34–36	654/81,574	1.19	1.04, 1.35	No full siblings in the data *n*= 578,936 (type 1 diabetes *n*= 3830)
	37–38	2763/365,452	1.11	1.04, 1.19	Final full sibling cohort *n*= 1,916,564 (76%)
	39–41	8637/1,322,338	1.00	[Reference]	
	42–45	764/128,493	0.97	0.87, 1.08	
	Total	12,977/1,916,564			
Pooled	<32	128/28,474	0.82	0.62, 1.09	
	32–33	199/30,335	1.09	0.86, 1.37	
	34–36	1306/175,697	1.08	0.99, 1.18	
	37–38	5610/752,736	1.11	1.06, 1.16	
	39–41	18,356/2,926,655	1.00	[Reference]	
	42–45	1490/265,131	0.96	0.89, 1.02	
	Total	27,089/4,179,028			

[a] HRs are adjusted for child's sex and birth year and mother's age, education level, parity, diabetes during pregnancy and hypertensive disorders during pregnancy (model 2)

ESM Table 13. Association between gestational age and the risk of type 1 diabetes – country-specific and pooled adjusted estimates using Maršál reference for calculating birthweight z scores

**Country**	**Gestational age category (weeks)**	**Type 1 diabetes, *n***	**Total, *n***	**Model Country: *n***	
				Model 2 FI: 1,652,345 NO: 1,322,124 SE: 2,498,229	
				HR[a]	95% CI
Finland	23–27	12	2678	0.85	0.48, 1.50
	28–31	51	8298	0.92	0.70, 1.21
	32–33	92	11,239	1.12	0.91, 1.38
	34–36	558	65,573	1.12	1.02, 1.22
	37–38	2588	293,144	1.16	1.11, 1.21
	39–41	8533	1,187,570	1.00	[Reference]
	42–45	495	71,514	0.98	0.89, 1.07
Norway	23–27	6	2153	0.69	0.31, 1.54
	28–31	24	8249	0.66	0.44, 0.98
	32–33	55	11,275	1.05	0.81, 1.38
	34–36	317	59,855	1.10	0.98, 1.23
	37–38	1160	213,125	1.15	1.11, 1.23
	39–41	4253	920,258	1.00	[Reference]
	42–45	449	100,945	0.86	0.78, 0.94
Sweden	23–27	16	4106	0.69	0.42, 1.12
	28–31	80	13,950	0.88	0.70, 1.09
	32–33	124	18,808	0.98	0.82, 1.17
	34–36	861	106,246	1.16	1.08, 1.25
	37–38	3558	462804	1.13	1.09, 1.17
	39–41	11,181	1,711,807	1.00	[Reference]
	42–45	942	163740	0.90	0.84, 0.96
Pooled	23–27	34	8937	0.74	0.53, 1.04
	28–31	155	30,497	0.85	0.73, 1.00
	32–33	271	41,322	1.04	0.92, 1.17
	34–36	1736	231,674	1.14	1.08, 1.19
	37–38	7306	969,073	1.14	1.12, 1.17
	39–41	23,967	3,819,635	1.00	[Reference]
	42–45	1886	336,199	0.91	0.87, 0.95

FI, Finland; NO, Norway; SE, Sweden

[a] HRs are adjusted for child's sex, birth year and birthweight z score calculated according to Maršál et al [23] and mother's age, education level, parity, diabetes during pregnancy and hypertensive disorders during pregnancy (model 2)

ESM Table 14 Country-specific and pooled associations of combinations of gestational age and birthweight z score according to Maršál reference with the risk of type 1 diabetes

Country	Gestational age category (weeks)	Birthweight z score category [a] Country: Type 1 diabetes/total, *n*											
		< -2		-2 to < -1		-1 to < 0		0 to< +1		+1 to < +2		≥ +2	
		FI: 327/58,247<br>NO: 219/54,404<br>SE: 316/51,213		FI: 1529/236,004<br>NO: 807/198,679<br>SE: 1713/282,483		FI: 3734/533,417<br>NO: 1895/425,827<br>SE: 5195/810,299		FI: 3978/511,617<br>NO: 1943/398,671<br>SE: 5919/836,001		FI: 2257/265,310<br>NO: 1134/201,802<br>SE: 2772/386,606		FI: 504/47,750<br>NO: 266/42,741<br>SE: 941/116,589	
		HR[b]	95% CI	HR[b]	95% CI	HR[b]	95% CI	HR[b]	95% CI	HR[b]	95% CI	HR[b]	95% CI
Finland	<32	0.62	0.37, 1.04	1.03	0.67, 1.59	0.78	0.48, 1.28	0.65	0.31, 1.37	c	c	0.92	0.34, 2.45
	32–33	0.63	0.35, 1.11	0.78	0.48, 1.25	1.32	0.94, 1.86	0.79	0.45, 1.39	2.72	0.86, 5.07	1.17	0.58, 2.35
	34–36	0.53	0.37, 0.76	1.07	0.88, 1.31	1.07	0.92, 1.26	1.10	0.93, 1.30	1.10	0.88, 1.38	1.27	0.95, 1.70
	37–38	0.89	0.72, 1.10	0.92	0.82, 1.05	1.07	0.99, 1.17	1.14	1.05, 1.24	1.25	1.15, 1.37	1.17	0.99, 1.39
	39–41	0.80	0.69, 0.94	0.87	0.81, 0.94	0.92	0.87, 0.97	1.00	[Reference]	1.01	0.95, 1.08	1.10	0.97, 1.25
	42–45	0.66	0.37, 0.17	0.84	0.68, 1.04	0.90	0.78, 1.05	1.01	0.86, 1.19	0.97	0.73, 1.29	0.89	0.50, 1.62
Norway	<32	0.50	0.25, 1.00	0.37	0.15, 0.88	0.90	0.51, 1.58	0.49	0.15, 1.51	c	c	0.57	0.14, 2.29
	32–33	0.50	0.22, 1.11	1.06	0.64, 1.77	0.89	0.52, 1.51	0.85	0.42, 1.69	1.66	0.62, 4.43	1.50	0.75, 3.00
	34–36	1.03	0.73, 1.44	0.83	0.62, 1.11	0.98	0.78, 1.22	1.25	1.01, 1.56	0.96	0.70, 1.31	0.97	0.66, 1.42
	37–38	0.74	0.54, 1.03	0.87	0.73, 1.05	1.07	0.95, 1.21	1.09	0.97, 1.23	1.34	1.18, 1.53	1.24	0.98, 1.58
	39–41	0.74	0.61, 0.90	0.82	0.74, 0.91	0.90	0.83, 0.97	1.00	[Reference]	1.08	0.99, 1.18	1.03	0.87, 1.23
	42–45	0.80	0.54, 1.18	0.71	0.57, 0.89	0.79	0.67, 0.94	0.80	0.67, 0.96	0.86	0.64, 1.16	0.85	0.50, 1.44
Sweden	<32	0.58	0.35, 0.96	1.30	0.91, 1.85	0.55	0.34, 0.87	0.65	0.41, 1.05	1.65	0.23, 11.70	0.94	0.56, 1.60
	32–33	0.74	0.45, 1.24	0.80	0.51, 1.25	0.99	0.71, 1.37	0.80	0.54, 1.19	1.10	0.55, 2.21	1.32	0.86, 2.03
	34–36	0.58	0.41, 0.81	0.95	0.78, 1.16	1.04	0.91, 1.20	1.12	0.98, 1.27	1.54	1.34, 1.78	1.27	1.03, 1.57
	37–38	0.66	0.51, 0.85	1.00	0.89, 1.12	1.06	0.98, 1.14	1.15	1.08, 1.23	1.18	1.10, 1.27	1.13	1.00, 1.29
	39–41	0.93	0.81, 1.07	0.88	0.82, 0.94	0.93	0.89, 0.97	1.00	[Reference]	1.03	0.98, 1.08	1.04	0.95, 1.14
	42–45	1.14	0.82, 1.59	0.78	0.65, 0.93	0.83	0.74, 0.94	0.89	0.79, 1.00	0.92	0.77, 1.10	0.79	0.57, 1.09
Pooled	<32	0.58	0.42, 0.79	1.07	0.82, 1.39	0.71	0.53, 0.95	0.63	0.43, 0.92	1.65	0.23, 11.77	0.89	0.57, 1.38
	32–33	0.65	0.46, 0.92	0.86	0.65, 1.13	1.09	0.88, 1.36	0.81	0.60, 1.08	1.58	0.98, 2.54	1.32	0.96, 1.93
	34–36	0.69	0.56, 0.84	0.97	0.86, 1.10	1.04	0.95, 1.14	1.14	1.04, 1.25	1.33	1.19, 1.49	1.22	1.04, 1.42
	37–38	0.78	0.67, 0.90	0.95	0.88, 1.02	1.07	1.01, 1.12	1.14	1.09, 1.19	1.23	1.17, 1.29	1.16	1.06, 1.27
	39–41	0.84	0.77, 0.92	0.87	0.83, 0.91	0.92	0.89, 0.95	1.00	[Reference]	1.03	1.00, 1.07	1.06	0.99, 1.13
	42–45	0.92	0.73, 1.16	0.78	0.69, 0.87	0.84	0.78, 0.91	0.90	0.83, 0.98	0.92	0.80, 1.05	0.82	0.64, 1.05

FI, Finland; NO, Norway; SE, Sweden

[a] Birthweight z scores calculated according to Maršál et al intrauterine growth reference [23] [b] HRs are adjusted for child's sex and birth year and mother's age, education level, parity, diabetes during pregnancy and hypertensive disorders during pregnancy (model 2) [c] Not enough individuals for the analyses

ESM Table 15 Country-specific associations between gestational age week by week and the risk of type 1 diabetes

Gestational week	Country											
	Finland				Norway				Sweden			
	Type 1 diabetes, *n*	Total, *n*	HR [a]	95% CI	Type 1 diabetes, *n*	Total, *n*	HR [a]	95% CI	Type 1 diabetes, *n*	Total, *n*	HR [a]	95% CI
23–28	14	4687	0.71	0.32, 1.13	6	3737	0.35	0.16, 0.79	28	7192	0.69	0.44, 1.45
29	8	1593	0.76	0.38, 1.52	<5	1553	0.53	0.20, 1.42	14	2876	0.72	0.43, 1.22
30	13	2214	0.86	0.50, 1.48	8	2218	0.76	0.38, 1.53	25	3820	0.97	0.66, 1.44
31	18	2911	0.87	0.54, 1.38	12	2898	0.85	0.48, 1.51	29	5056	0.84	0.59, 1.22
32	24	4359	0.76	0.51, 1.14	23	4358	1.10	0.73, 1.66	46	7512	0.91	0.68, 1.22
33	66	6553	1.39	1.09, 1.77	32	6536	0.99	0.70, 1.40	77	11,327	1.00	0.80, 1.25
34	94	10,958	1.20	1.09, 1.47	61	11,010	1.11	0.86, 1.44	150	18,753	1.18	1.00, 1.38
35	166	18,768	1.19	1.02, 1.40	77	17,350	0.89	0.71, 1.12	225	30,974	1.06	0.93, 1.22
36	297	36,230	1.09	0.96, 1.23	182	31,702	1.16	1.00, 1.36	490	57,623	1.24	1.13, 1.36
37	777	81,658	1.25	1.15, 1.35	374	64,286	1.21	1.08, 1.36	1085	129,510	1.24	1.16, 132
38	1810	214,047	1.18	1.11, 1.25	786	149,985	1.12	1.03, 1.22	2473	336,856	1.13	1.07, 1.18
39	3179	415,614	1.10	1.04, 1.15	1356	282,586	1.04	0.96, 1.11	3996	571,026	1.10	1.05, 1.14
40	3287	472,401	1.00	[Reference]	1719	369,290	1.00	[Reference]	4467	700,428	1.00	[Reference]
41	2067	308,044	0.98	0.92, 1.03	1178	272,340	0.92	0.86, 0.99	2726	451,551	0.96	0.91, 1.00
42	495	72,006	0.99	0.90, 1.09	447	101,214	0.82	0.74, 0.91	942	164,610	0.90	0.84, 0.97
43–44	7	1343	0.58	0.27, 1.21	99	16,626	0.92	0.75, 1.12	83	11,077	0.92	0.74, 1.14

[a] HRs are adjusted for child's sex and birth year and mother's age, education level, parity, diabetes during pregnancy and hypertensive disorders during pregnancy (model 2)

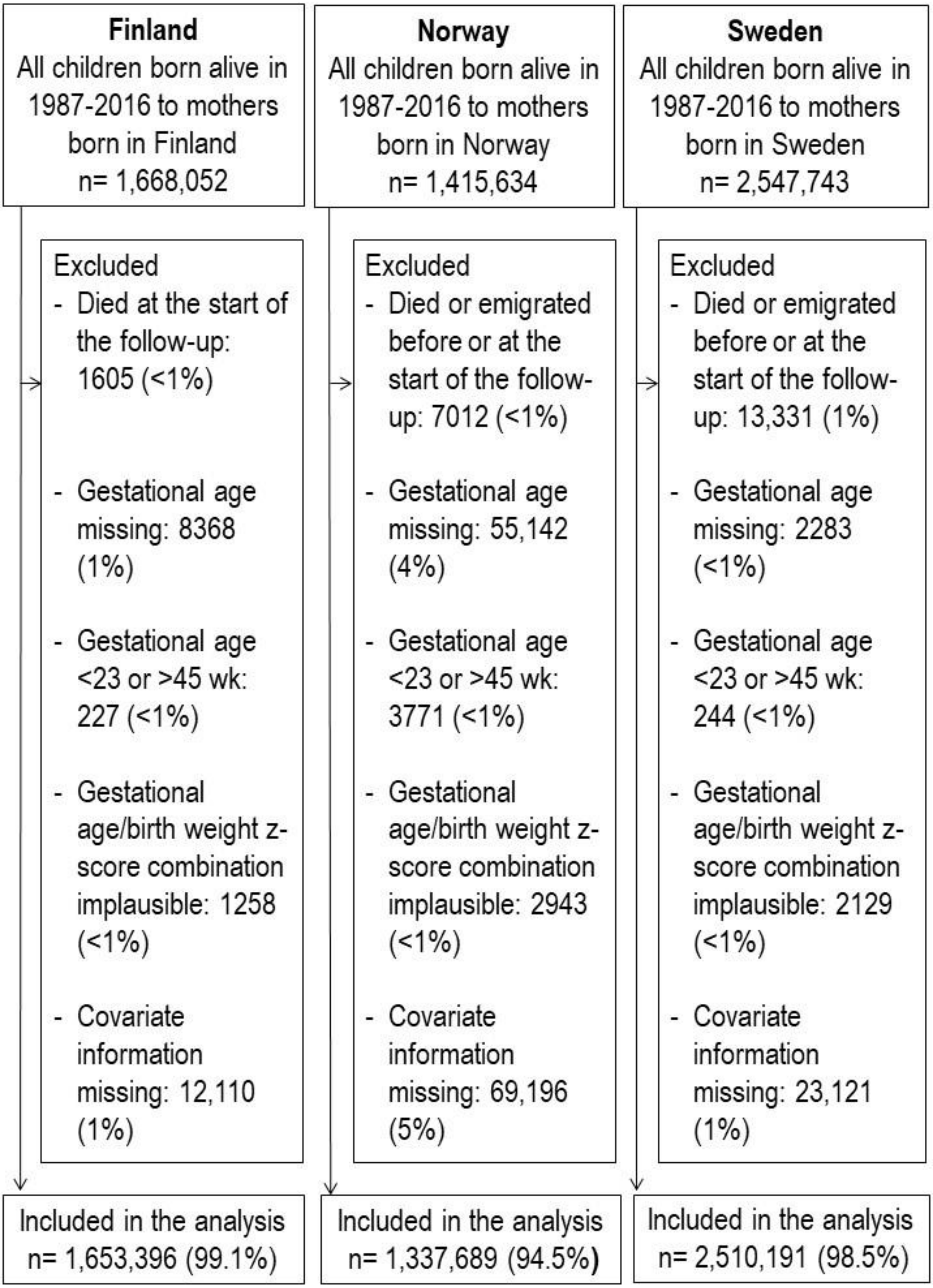

ESM Fig. 1 Flow chart of the study participants

A)

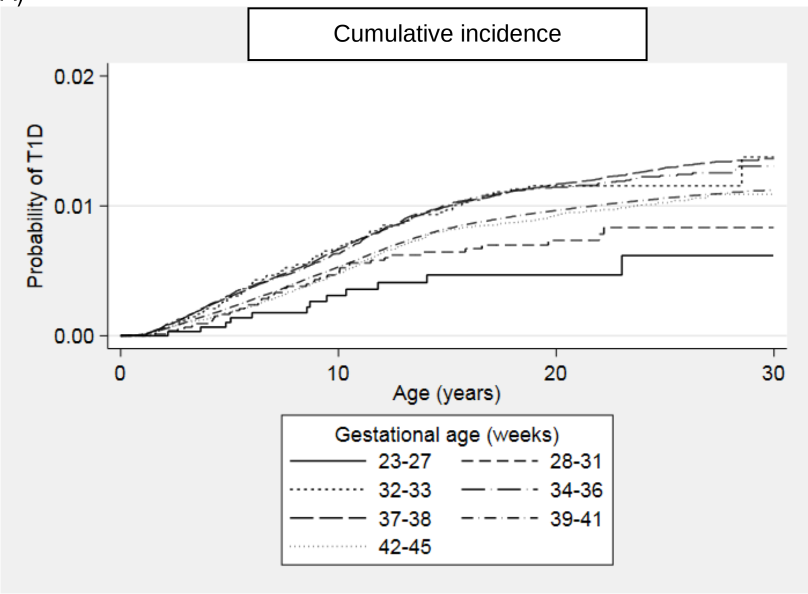

B)

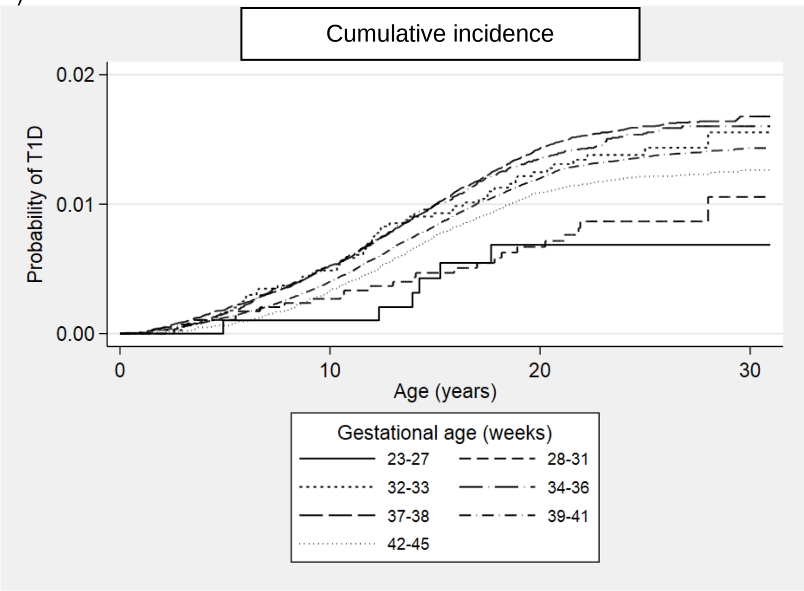

C)

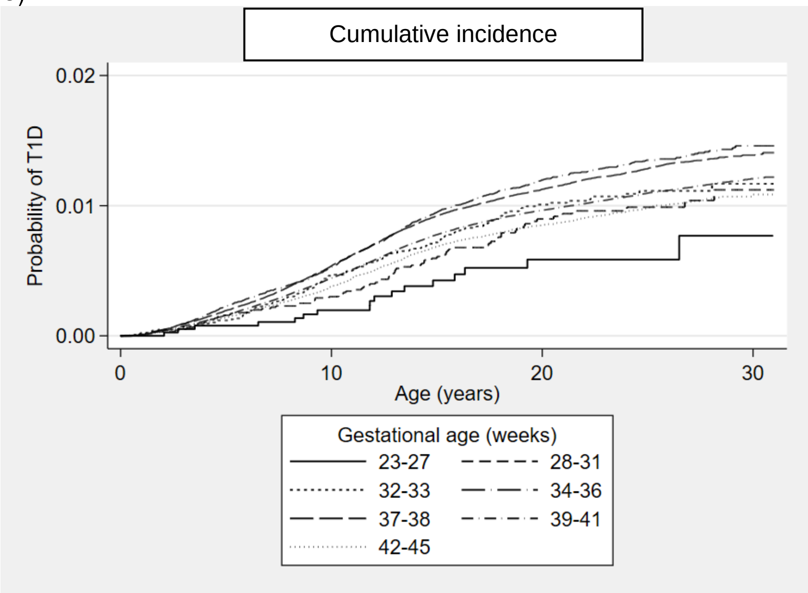

ESM Fig. 2. Cause-specific cumulative incidence of type 1 diabetes in gestational age categories in A) Finland, B) Norway, and C) Sweden

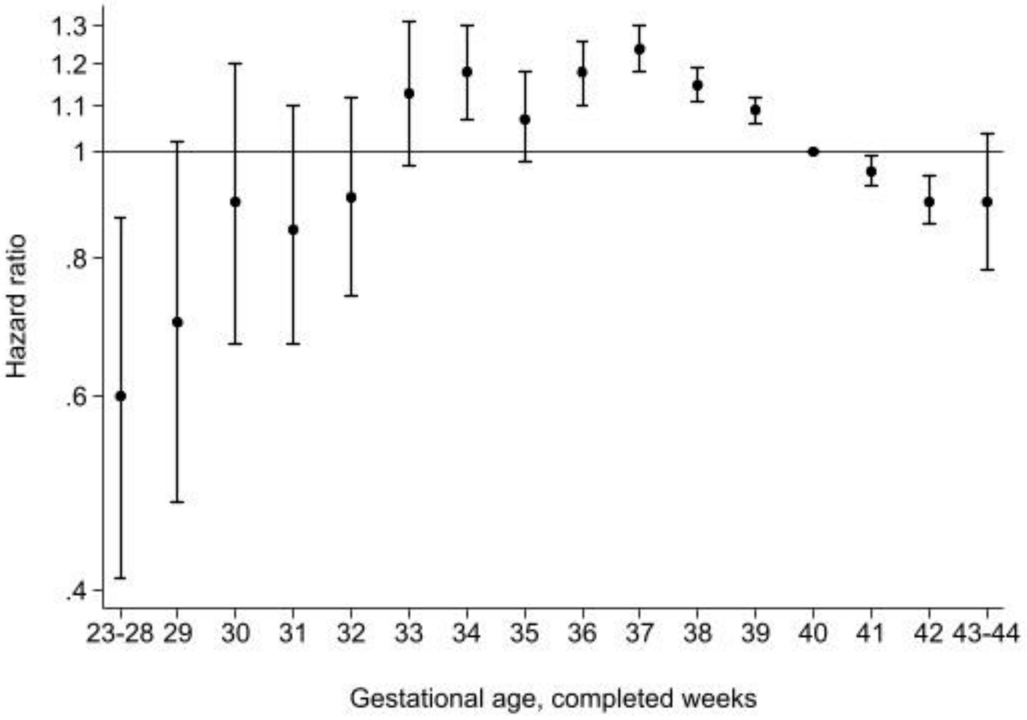

ESM Fig. 3. Association between gestational age in weeks and risk of type 1 diabetes in offspring in Finland, Norway and Sweden. Gestational weeks 23–28 and 43–44 are combined and week 40 is the reference category. Pooled HRs are adjusted for child's sex, birth year and birthweight z score and mother's age, education level, parity, diabetes during pregnancy and hypertensive disorders during pregnancy (model 2).
